# A comprehensive survey of scoring functions for protein docking models

**DOI:** 10.1186/s12859-024-05991-4

**Published:** 2025-01-22

**Authors:** Azam Shirali, Vitalii Stebliankin, Ukesh Karki, Jimeng Shi, Prem Chapagain, Giri Narasimhan

**Affiliations:** 1 Bioinformatics Research Group (BioRG), Knight Foundation School of Computing and Information Sciences, Florida International University, 11200 SW 8th 10 St, Miami, 33199 USA; 2 Department of Physics, Florida International University, 11200 SW 8th 10 St, Miami, 33199 USA; 3 Biomolecular Sciences Institute, Florida International University, 11200 SW 8th St, Miami, 33199 USA

**Keywords:** Computational structural biology, Protein-protein interactions, Scoring functions, Molecular docking, Deep learning, Protein surface properties

## Abstract

**Background:**

While protein-protein docking is fundamental to our understanding of how proteins interact, scoring protein-protein complex conformations is a critical component of successful docking programs. Without accurate and efficient scoring functions to differentiate between native and non-native binding complexes, the accuracy of current docking tools cannot be guaranteed. Although many innovative scoring functions have been proposed, a good scoring function for docking remains elusive. Deep learning models offer alternatives to using explicit empirical or mathematical functions for scoring protein-protein complexes.

**Results:**

In this study, we perform a comprehensive **survey** of the state-of-the-art scoring functions by considering the most popular and highly performant approaches, both classical and deep learning-based, for scoring protein-protein complexes. The methods were also compared based on their runtime as it directly impacts their use in large-scale docking applications.

**Conclusions:**

We evaluate the strengths and weaknesses of classical and deep learning-based approaches across seven public and popular datasets to aid researchers in understanding the progress made in this field.

**Supplementary Information:**

The online version contains supplementary material available at 10.1186/s12859-024-05991-4.

## Introduction

Protein-protein docking is key to understanding how proteins interact. Accurate and efficient scoring functions that differentiate between native and non-native binding complexes is critical for the accurate docking computations. The scoring task is highlighted in the challenge contest called CAPRI: Critical Assessment of PRediction of Interactions. The goal of this paper is to provide a practical comparison of the state-of-the-art scoring tools.

The field of structural biology has been advancing rapidly for the past few decades, and experimental approaches (such as nuclear magnetic resonance (NMR), X-ray crystallography, and cryogenic electron microscopy) have enabled biologists to determine the 3D structures of many proteins that have been published in Protein Data Bank [1]. However, almost all biological functions in living organisms depend on the interactions between proteins, and determining the structure of complexes resulting from these interactions is essential for drug discovery and therapeutic development. As with individual proteins, experimental approaches to determine the structures of complexes do exist but are costly and time-consuming, thus motivating the need for fast and accurate computational methods [2].

Computational docking methods predict the 3D structure of a complex using the 3D structures of individual proteins. These methods typically involve two steps. The first step is *sampling*, during which numerous candidate conformations are generated. Following this, a *scoring* step is performed to identify the conformations closest to the native structure. A good scoring function should correctly and efficiently score the near-native models highly, and assign low scores to the non-native conformations. Advances in computing hardware have greatly improved the first step of docking and have been surveyed by Vakser et al. [3]. However, as mentioned above, accurate scoring functions remain a challenge and consequently, the accuracy of docking tools cannot be guaranteed. Applications of scoring and docking include drug and vaccine design, where it helps virtually predict if and how proteins may react to other molecules, thus saving time and resources that would otherwise be spent on experimental screening. As a result, the reliability of scoring functions directly affects the success rate of therapeutics. Consequently, scoring functions are crucial in improving computational docking tools and accelerating the development of new pharmaceuticals and vaccines. This comprehensive survey will review the literature on *scoring functions* for protein-protein docking.

Note that this survey will **not** cover docking methods since they have been adequately discussed elsewhere. Assessment of the strengths and weaknesses of various docking methods and their range of applications are discussed in [4–6]. Additionally, several authors have examined the challenges and limitations of molecular docking methods, and have surveyed the shortcomings and unanswered questions within the field of molecular docking [2, 7–10]. Many surveys of docking tools for drug discovery exist in the literature [11–13], reviewing protein-ligand docking tools. This survey will not review methods for *docking* or protein-ligand docking, but will focus on scoring functions for protein-protein docking.

**Fig. 1:**
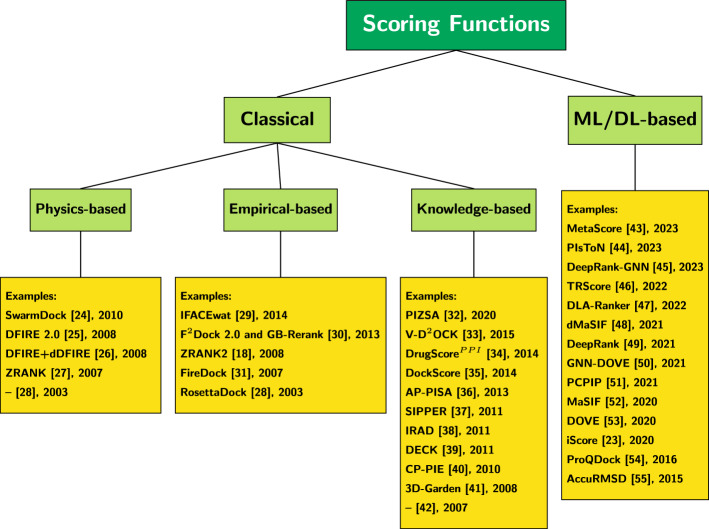
Categories of scoring functions for protein-protein docking models and relevant publications

Scoring functions can be divided into four categories: (1) physics-based, (2) empirical-based, (3) knowledge-based, and 4) those based on machine learning (ML) or deep learning (DL) [14]. The introduction of *physics-based* scoring functions began with classical force field methods that calculated binding energy by summing the Van der Waals and electrostatic interactions between two proteins [14]. Scoring functions were further improved by introducing solvent effects, polarization, and charge features [14]. However, the bottom line is that all physics-based models have high computation costs [14]. *Empirical-based* methods estimate the binding affinity of a complex, which is the difference of free energy upon complex formation. This is done by summing up a series of weighted energy terms before and after bonding. As these binding affinities are simulated using data from already known 3D structures, their scoring functions are simpler and more straightforward to compute compared to those in physics-based methods [15], making empirical-based methods faster than physics-based methods. The *knowledge-based*, or statistical-potential scoring functions use the pairwise distances between atoms or residues in the two proteins and convert them into potentials through Boltzmann inversion [16]. Knowledge-based scoring functions offer a good balance between accuracy and speed [17]. Hybrid approaches for scoring have also been proposed [18–22]. With major advances in ML and DL, it is not surprising that many learning models exist for estimating scoring functions. ML and DL approaches can learn complex transfer functions that map a combination of interface features, energy, and accessible surface area terms to predict scoring functions [23]. Of the four categories of scoring functions, the first three are called *classical* methods. Figure 1 illustrates these categories and some of the publications in each.

A categorization of ML- and DL-based scoring functions was presented by Li et al. [14] and reviewed by Huang et al., and Wang et al. [17, 56]. In [57], authors compared the performance of scoring functions on different types of complexes such as antibody-antigen, enzyme-inhibitor, etc. [58, 59] provide a comprehensive comparison of ML-based scoring functions for virtual screening.

Moal et al. [60] evaluated 115 different scoring functions, and Su et al. [61] provided a comparative assessment of scoring functions. However, these studies did not include ML-based efforts in molecular modeling. Moreover, advancements in ML and DL for protein-protein interaction analysis and molecular docking were reviewed recently, but without comparing them with non-ML methods [62, 63]. It is essential to compare and evaluate classical and modern efforts together, not separately. Furthermore, all scoring functions were benchmarked on different datasets and have not undergone a consistent, head-to-head comparison. This raises troubling questions about whether these tools are fine-tuned and tested on specific “in-distributions” and whether they would perform as well with other “out-of-distributions” datasets [64]. Our study bridges these two gaps by conducting a comprehensive comparison across seven public and popular datasets.

The following is the organization of this paper. Section Methods summarizes all methods being compared. In Sect. Datasets, we introduce the different datasets used in this work. The results of the experiments in Sect. Results are followed by a discussion section.

## Methods

This study compares the performance of eight commonly used classical methods (or their hybrids) with four cutting-edge DL-based methods. Below, we briefly summarize these methods and their properties.

### Classical methods

We start with a brief description of the classical methods for scoring complexes.

**FireDock**: [31] Calculates the free energy change of the residues at the interface of a complex, uses an SVM [65] to calibrate the weights, and calculates the linear weighted sum of some energy terms as the binding score. After the refinement step, refined conformations are scored by calculating free energy contributions from desolvation, electrostatics, internal energies (bond stretch, angle, and torsion), hydrogen and disulfide bonds, and van der Waals interactions.
**PyDock**: Uses a scoring function that balances the electrostatic and desolvation energies [66]. It uses electrostatic energies with a distance-dependent dielectric constant and weighted desolvation energies for the score.
**RosettaDock**: Scores the final refined complexes by minimizing an energy function that sums up contributions from van der Waals forces, hydrogen bonds, electrostatics, solvation, and side chain rotamer energies [28]. Our experiments were conducted with PyRosetta [67], a Python-based implementation of Rosetta v3.1.
**ZRANK2**: [18], An extension of ZRANK [27], calculates a linear weighted sum of energy terms representing van der Waals energy, electrostatics (attractive, repulsive, short-range, and long-range), and desolvation (using pairwise Atomic Contact Energy (ACE)). It employs RosettaDock to refine each model to generate 300 structures per predicted complex.
**AP-PISA**: [36] Uses a distance-dependent pairwise atomic potential in combination with a residue potential to rescore the refined complexes. The two potentials have different signals, thus increasing the chance of generating correct solutions.
**CP-PIE**: [40] Finds the overlap and solvent-accessible surface areas at the complex interface. They argue that the total overlap area for correct complexes is less than that for incorrect complexes and use it as a filter to eliminate candidate complexes. The binding score also uses the number of residue contacts.
**SIPPER**: [37] Uses a combination of residue-residue interface propensities for each possible residue pair and a residue desolvation energy based on solvent-exposed area for scoring predicted complexes.
**HADDOCK**: [68] Scores the docking models using several energetic and empirical criteria. It includes terms for Van der Waals forces, electrostatic interactions, and desolvation energy. In addition to these energetic considerations, HADDOCK’s scoring function also takes into account the degree to which a docking model adheres to or violates the experimental data that were used to guide the docking process, such as residues on the interface, solvent accessibility, intermolecular distance between atoms of both proteins that participate in the interaction. Our experiments were conducted with HADDOCK v3 [69].

Among these methods, FireDock, RosettaDock, and ZRANK2 are empirical-based methods. AP-PISA, CP-PIE, and SIPPER are knowledge-based methods, while PyDock and HADDOCK are hybrid methods that combine elements from the categories described in Introduction section. The different docking tools and strategies used by each method are summarized in Table S1.

In this survey, our focus is to evaluate the scoring methods only, without taking into account the docking process. Therefore, we utilized the CCharPPI server [70], which allows us to assess only the scoring functions independent of docking and other components that some of the tools may have.

Ten other classical methods that are primarily focused on docking include HDOCK [71], ClusPro [72], ZDOCK [73], PatchDock [74], SWARMDOCK [75], ATTRACT [76], GRAMM-X [77], 3D-Garden [41], InterEvDock [78], and HEX [79]. They were not included in our comparison because they package the docking and scoring steps together, with no provision for a separate module for scoring. Consequently, we are not aware of a way to score pre-docked models with these tools, making it impossible for us to isolate their performance on scoring.

### Deep learning-based methods

Deep learning (DL) has been successfully applied across a wide range of applications and data modalities, including text [80, 81], images [82–84], graphs [85, 86], and time series data [87–90]. DL has also been applied to various problems in the field of protein science. Here, we survey four DL-based approaches for scoring functions.

**GNN-DOVE**: Stands for Graph Neural Network (GNN)-based DOcking decoy eValuation scorE [50], and is an extension of DOVE [53]. It defines two subgraphs to represent the interface of the complex. Physicochemical features, such as the type of the atom, the number of connections, and the number of attached Hydrogen atoms, are incorporated as node features in the graph using one-hot encodings. Edges represent covalent and non-covalent bonds. A gate-augmented attention mechanism is used to learn the pattern of atomic interactions at the interface and to assess the importance of each interaction.
**DeepRank-GNN**: [45] Is also a GNN-based approach that learns “embeddings” of a complex. It is an extension of DeepRank [49] and represents the interface of the complex as a graph using residue-level features like type, charge, and buried surface area (BSA), for each node. Residue-level features are represented as node features. The graph representation has two subgraphs, one for the connections between residues within each protein and the other showing the connections between residues of the two interacting proteins. The subgraphs are then passed separately to two convolution layers, one for a graph attention network (GAT) [91] and the other for edge aggregated graph attention network (EGRAT) [92]. Using these layers, the contributions of the neighbors to each node in subgraphs are calculated and weighted. The weighted sum of the neighbors’ feature representation as an aggregator helps the network to distinguish native complexes from non-native complexes.
**dMaSIF**: (Differentiable Molecular Surface Interaction Finger-printing) [48], unlike its predecessor, MaSIF [52], represents the protein surface as a point cloud and determines the interactions between all atoms of the complex without using precomputed interface features. For each point of the complex interface, it calculates ten geometric features and six chemical features. These features are then passed to a sequence of (quasi-)geodesic convolutional layers for learning. The resulting binding score from dMaSIF is fast and accurate.
**PIsToN**: (Protein Interfaces with Transformer Network) [44] is the most recent of the DL-based tools. PIsToN crops and isolates pairs of patches from the interface, one for each interacting protein. The information from each patch is then converted into multichannel images, with each channel representing one of the features of the interaction, including Relative Accessible Surface Area (RASA), curvature, charge, electrostatics, and hydrophobicity. PIsToN has three components. The use of a Vision Transformer (ViT) [93] differentiates it from the other methods. The embedding produced by the ViT is then combined with “hybrid” empirical energy terms, including Van der Waals, electrostatics, and desolvation, allowing it to learn the characteristics of complex binding energies. Finally, a multi-attention model groups energy terms and interface features into five groups, each containing an independent transformer network. These independent networks are aggregated into a transformer encoder to score the complexes as native or non-native decoys.

It is important to note that the aforementioned DL-based methods have predecessor versions, which were not included in our study because their performance was shown to be lower than their corresponding successors. Therefore, MaSIF [52], DOVE [53], and DeepRank [49] were excluded from our comparisons. Additionally, all DL-based scoring functions for protein-ligand complexes were deemed to be outside the scope of the current study and excluded as well.

## Datasets

Seven different datasets were used in this work to evaluate the methods. Links to access to these datasets are provided in the Data Availability section. Since each data set may represent widely different protein complexes and significantly different distributions, the use of these multiple data sets is particularly important to shed light on the generalizability of the DL-based methods, which may be prone to training set biases.

**CAPRI Score v2022**: Refers to the dataset used for the Critical Assessment of Predicted Interactions (CAPRI) competition that invites submissions of computational methods to compete based on a set of protein complexes [94]. Since 2005, CAPRI has provided improved datasets for independent testing of new scoring functions apart from docking [95]. Unlike the other datasets in this section, the CAPRI Score v2022 dataset was generated by a variety of different docking tools resulting in a diverse range of models, making it an excellent dataset for the evaluation of scoring functions. This dataset contains all published CAPRI complexes (up to CAPRI Round 50 and joint CAPRI/CASP Rounds). It contains 96 complexes and 148 interfaces with a total of 170,310 docking models of varying complexities. After discarding multi-protein [96] complexes and the complexes for which there is no correct solution, we had 44 difficult and 39 easy complexes with a total of 80,321 docking models. The level of difficulty for a complex is determined based on the similarity of its sequence and structure to other proteins with known experimental structures [97]. In our experiments, we treated the difficult complexes and easy complexes as separate datasets to more precisely compare the performance of the methods. Henceforth, we will refer to the two data sets as **CAPRI Score v2022 Difficult** (44 complexes with 39,130 docking models) and **CAPRI Score v2022 Easy** (39 complexes with 41,191 models). These complexes, with all their models and levels of difficulties, are available on Zenodo at https://zenodo.org/records/12681335.
**CAPRI Score Refined**: Refers to another dataset called CAPRI Score [95] (not v2022) containing 13 complexes used in CAPRI rounds 13 [98] through 26 [99] with roughly 500 to 2,000 docking models per target for a total of 16,666 docking models of varying complexity, which were refined using the HADDOCK docking tool [68]. This dataset was introduced in the DeepRank [49] paper.
**Dockground**: Dataset 1.0 was generated using Gramm-X [100] and contains 61 complexes. Each complex has 100 non-native and at least one near-native docking model (on average, 9.83 near-native docking models per complex). There are a total of 6,725 docking models in the dataset.
**BM4**: Benchmark 4.0 [101] contains 176 unbound complexes. Each protein is available in a bound and an unbound conformation. Each complex has 54,000 docking models. For each complex, we subsampled the 400 top predictions (each complex has at least one near-native docking model). The GNN-DOVE [50] study used this dataset as its training set and left 19 complexes as a test set. To make the comparison fair, we used the 19 complexes as a test set in our experiments, which provided a total of 7,600 docking models. These chosen complexes are available on Zenodo at https://zenodo.org/records/12681335.
**BM5**: Dataset contains 231 high-quality complexes. For each complex, the bonded and unbound conformations of the proteins involved in the complex are available [102]. BM5 augments BM4, which had 55 complexes. The DeepRank-GNN [45] study generated 25,300 docking models for each complex using the HADDOCK software [68]. We randomly subsampled 500 docking models for each of the 15 complexes that were not used in training or validating DeepRank-GNN, resulting in 7,500 docking models in total. These chosen complexes are available on Zenodo at https://zenodo.org/records/12681335.
**PDB-2023**: Was used in the PIsToN paper [44] and contains complexes that are deposited in the RCSB Protein Data Bank (PDB) [1] in the year 2023. All docking models were generated using HDOCK [71]. This dataset contains models for 53 heterodimers, with 126 near-native and 5174 non-native models in total.
**MaSIF dataset**: Was used for training and testing the MaSIF-search tool [52]. Later, the PIsToN work used 678 complexes from this dataset as a testing set, and for each complex, randomly used one non-native and one near-native conformation [44]. We used exactly this test dataset for our experiments here.

## Results

In this section, we discuss the performance of all 12 methods on all seven datasets. As mentioned in Datasets section, we divided the CAPRI Score v2022 dataset into two subsets: difficult complexes and easy complexes. Consequently, in this section, we have the results of each method on eight datasets.

For DL-based methods, we used the best pretrained model, which had the best performance according to the authors’ recommendations in their corresponding paper or GitHub page. Also, we followed the settings mentioned below for each method. For GNN-DOVE, we chose fold model 5 which had the best performance. For DeepRank-GNN, we used the PSSMGen [103] tool to compute Position-Specific Scoring Matrices (PSSM) features. For PIsToN, we chose a patch size of 16 Å, since the authors mentioned in their paper that this patch size provides the best performance. We calculated the average, minimum, and maximum scores for all potential contacts in dMaSIF. As the average scores had the highest AUC on all datasets, we reported them in the results. For the classical methods, we used the CCharPPI server [70], which computes the values simultaneously. No pre-calculated features and setting parameters are needed to use this server.

*Labeling the data:* The CAPRI-Q tool [104] is used to assess the quality of the docking models and categorize them based on the CAPRI criteria [105]. The CAPRI-Q classifies docking models into four categories: incorrect, acceptable, medium, or high, according to their quality. So, a docking model is considered **incorrect** if:

$$\begin{aligned} (\text {fnat} < 0.1) \quad {\textbf {OR}} \quad (\text {lRMSD}> 10 \, \text{\AA }\quad \text {and} \quad \text {iRMSD} > 4 \, \text{\AA }). \end{aligned}$$

Otherwise, the quality can be **acceptable**, as defined below:

**Acceptable quality:**

$$\begin{aligned} \hspace{2em} (\text {fnat} \ge 0.1 \quad \text {and} \quad \text {lRMSD} \le 10 {\text{\AA }})\quad {\textbf {OR}} \quad (\text {fnat} \ge 0.1 \quad \text {and} \quad \text {iRMSD} \le 4 {\text{\AA }}),\quad \end{aligned}$$

or **medium quality**, as defined below:

$$\begin{aligned} (\text {fnat} \ge 0.3 \quad \text {and} \quad \text {lRMSD} \le 5 {\text{\AA }})\quad {\textbf {OR}} \quad (\text {fnat} \ge 0.3 \quad \text {and} \quad \text {iRMSD} \le 2 {\text{\AA }}), \quad \end{aligned}$$

or **high quality**, as defined below:

$$\begin{aligned} (\text {fnat} \ge 0.5 \quad \text {and} \quad \text {lRMSD} \le 1 {\text{\AA }})\quad {\textbf {OR}} \quad (\text {fnat} \ge 0.5 \quad \text {and} \quad \text {iRMSD} \le 1 {\text{\AA }}),\quad \end{aligned}$$

where *fnat* is the fraction of native contacts recovered, *iRMSD* is the Root Mean Square Deviation (RMSD) between the $C_{\alpha }$ atoms of the docked model and the native structure at the interface, and *lRMSD* is the global RMSD between the $C_{\alpha }$ atoms of the two structures. We labeled all acceptable, medium, and high categories as correct docking models.

### Comparing the AUC ROC measures

**Fig. 2:**
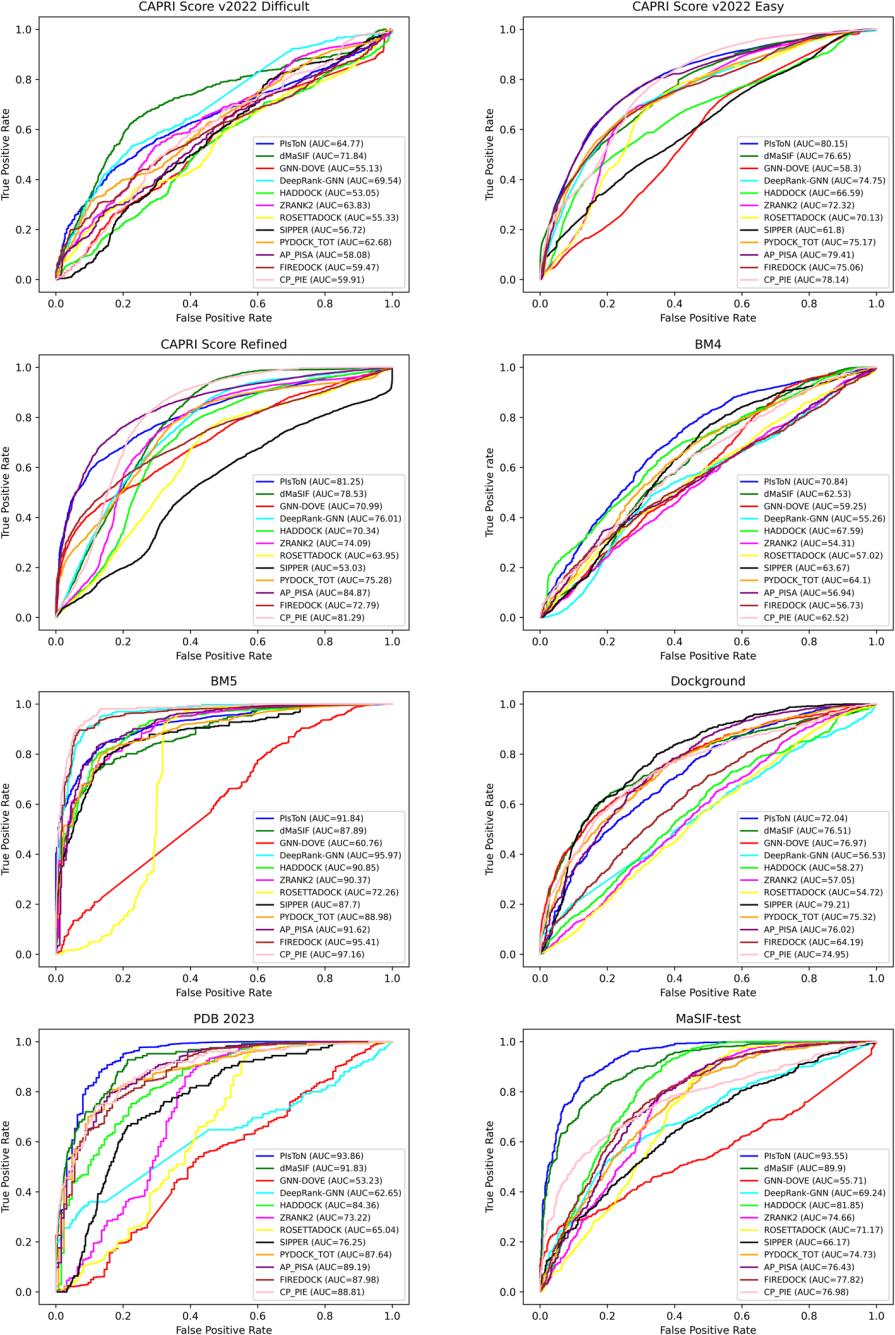
AUC ROC graphs of all twelve scoring functions on eight different datasets. The first four methods are DL-based, and the rest are classical methods (details are provided in Methods section)

To evaluate the performance of the methods, the area under the curve of the receiver operating characteristic (AUC ROC) was calculated (see Fig. 2). The ROC curve plots the fraction of true positives (TP) versus false positives (FP) while navigating the rankings provided by the scoring function. An ideal AUC ROC value is equal to 1, and a random 2-class classifier achieves 0.5. Additionally, various standard classification metrics are calculated, including the F1 score, precision, average precision (AP), recall, and balanced accuracy (BA) (see Supplemental Table S2).

Table 1 summarizes the AUC ROC of all the scoring functions on the eight datasets. In two out of eight datasets, the classical methods outperformed the DL-based methods. For the CAPRI score refined dataset, AP-PISA has the highest AUC value at 84.87. For the BM5 dataset, CP-PIE performed best with an AUC value of 97.16, and DeepRank-GNN is the second best with an AUC value of 95.97. It is worth noting that DeepRank-GNN used this dataset for training and validation, but only those not in training and validation sets were used in this comparison (see Datasets) section. It is worth mentioning that among classical tools, AP-PISA and CP-PIE were the best, with the highest AUC values for five out of eight datasets. HADDOCK performed better than other classical methods for the BM4 and MaSIF-test datasets, with AUC values of 67.59 and 81.85, respectively. For the remaining datasets, ZRANK2 was the best for the CAPRI score v2022 Difficult dataset, and SIPPER was the best for the Dockground dataset.

DL methods performed significantly better than classical methods for the remaining six datasets, namely, CAPRI Scoreset v2022 (both difficult and easy complexes), BM4, Dockground, PDB-2023, and MaSIF-test. PIsToN had the highest AUC value for four of the six datasets, while GNN-DOVE outperformed its competitors for Dockground. We note that GNN-DOVE applied 4-fold cross-validation on this dataset for its training, validation, and test parts. Scores obtained from each of the four test sets were used for a fair comparison. For CAPRI Scoreset v2022 difficult complexes, dMaSIF outperformed other tools with an AUC value of 71.84. For PDB-2023 and MaSIF-test dataset, PIsToN had significantly better performance than its closest competitor, dMaSIF, with AUC values of 93.86 and 91.83 for PDB-2023 and 93.55 and 89.90 for MaSIF-test dataset, respectively. We conclude that DL-based methods outperformed classical methods on six of the eight datasets.

**Table 1:** AUC ROC of all twelve scoring functions on eight different datasets

Approach	Method	CAPRI Score v2022 Difficult	CAPRI Score v2022 Easy	CAPRI Score Refined	BM4	BM5	Dockground	PDB-2023	MaSIF-test
Deep Learning	DeepRank-GNN	69.54	74.75	76.01	55.26	95.97	59.76	62.65	69.24
	GNN-DOVE	55.13	58.30	70.99	59.25	60.76	**83.87**	53.23	55.71
	dMaSIF	**71.84**	76.65	78.53	62.53	87.89	79.94	91.83	89.90
	PIsToN	64.77	**80.15**	81.25	**70.84**	91.84	68.42	**93.86**	**93.55**
Classical	FireDock	59.47	75.06	72.79	56.73	95.41	56.08	87.98	77.82
	AP-PISA	58.08	79.41	**84.87**	56.94	91.62	79.19	89.19	76.43
	CP-PIE	59.91	78.14	81.29	62.52	**97.16**	72.26	88.81	76.98
	PyDock	62.68	75.17	75.28	64.10	88.98	77.17	87.64	74.73
	ZRANK2	63.83	72.32	74.09	54.31	90.37	64.18	73.22	74.66
	RosettaDock	55.33	70.13	63.95	57.02	72.26	61.62	65.04	71.17
	SIPPER	56.72	61.80	53.03	63.67	87.70	79.60	76.25	66.17
	HADDOCK	53.05	66.59	70.34	67.59	90.85	53.15	84.36	81.85

Bold values indicate the best value for that column

### Comparing success rate measures

The second metric for evaluating tools is *success rate*, which measures how often a docking method provides at least one model of acceptable quality within its top 1, top 10, top 25, top 100, and top 200 predicted docking models. A higher success rate can impact the success of drug discovery efforts. The percentage of success rates of all twelve scoring functions on all datasets are shown in Table 2. As the MaSIF-test dataset has a unique property where each protein complex has only one correct and one incorrect docking model, the success rate metric could not be calculated for this dataset.

**Table 2:** Success rates of all twelve scoring functions on eight different datasets. The first four methods are DL-based, and the rest are classical methods (details in Methods) section

Dataset	Method	Top1	Top10	Top25	Top100	Top200
CAPRI Score v2022 Difficult (39,130 models)	DeepRank-GNN	14	**35**	33	65	78
	GNN-DOVE	0	7	28	42	57
	dMaSIF	14	32	**46**	**78**	**85**
	PIsToN	**17**	28	**46**	71	78
	FireDock	0	0	3	28	46
	AP-PISA	0	24	17	46	57
	CP-PIE	7	25	39	60	**85**
	PyDock	0	7	7	35	46
	ZRANK2	0	14	17	46	53
	RosettaDock	3	10	25	46	60
	SIPPER	3	14	35	53	60
	HADDOCK	3	14	21	46	60
CAPRI Score v2022 Easy (41,191 models)	DeepRank-GNN	35	79	82	92	**100**
	GNN-DOVE	15	48	56	76	84
	dMaSIF	**51**	71	**89**	**97**	**100**
	PIsToN	41	**82**	87	94	97
	FireDock	5	23	35	53	69
	AP-PISA	15	33	51	79	87
	CP-PIE	35	64	74	**97**	97
	PyDock	5	17	25	58	76
	ZRANK2	20	41	61	87	97
	RosettaDock	23	43	64	87	97
	SIPPER	25	58	74	92	**100**
	HADDOCK	15	69	71	84	97
CAPRI Score Refined (16,666 models)	DeepRank-GNN	23	46	53	69	84
	GNN-DOVE	15	61	69	**76**	**100**
	dMaSIF	15	38	53	**76**	92
	PIsToN	**38**	**69**	**76**	**76**	**100**
	FireDock	0	0	7	23	46
	AP-PISA	0	0	7	46	53
	CP-PIE	23	46	69	69	84
	PyDock	0	0	0	7	30
	ZRANK2	7	38	53	61	61
	RosettaDock	7	38	53	69	76
	SIPPER	7	46	53	69	76
	HADDOCK	7	23	53	69	76
BM4 (7,600 models)	DeepRank-GNN	21	73	73	89	94
	GNN-DOVE	21	68	84	**100**	**100**
	dMaSIF	47	68	89	94	**100**
	PIsToN	**54**	**89**	89	94	**100**
	FireDock	26	84	89	89	94
	AP-PISA	26	78	89	94	94
	CP-PIE	52	78	84	**100**	**100**
	PyDock	15	63	84	94	94
	ZRANK2	42	78	89	94	94
	RosettaDock	42	73	89	94	94
	SIPPER	47	63	68	78	94
	HADDOCK	26	**89**	**94**	94	**100**
BM5 (7,500 models)	DeepRank-GNN	**93**	**100**	**100**	**100**	**100**
	GNN-DOVE	6	26	33	53	73
	dMaSIF	40	80	**100**	**100**	**100**
	PIsToN	66	93	**100**	**100**	**100**
	FireDock	0	0	0	6	13
	AP-PISA	0	6	6	13	26
	CP-PIE	**93**	**100**	**100**	**100**	**100**
	PyDock	0	0	0	6	6
	ZRANK2	0	0	6	6	20
	RosettaDock	13	46	53	66	80
	SIPPER	60	93	93	**100**	**100**
	HADDOCK	0	0	0	6	26
Dockground (6,725 models)	DeepRank-GNN	37	53	72	**100**	**100**
	GNN-DOVE	**70**	**91**	**94**	**100**	**100**
	dMaSIF	34	75	87	**100**	**100**
	PIsToN	15	55	81	**100**	**100**
	FireDock	1	18	48	98	**100**
	AP-PISA	1	6	17	98	**100**
	CP-PIE	22	56	75	**100**	**100**
	PyDock	0	8	20	93	**100**
	ZRANK2	5	25	53	**100**	**100**
	RosettaDock	3	31	56	96	**100**
	SIPPER	43	72	89	**100**	**100**
	HADDOCK	10	44	65	**100**	**100**
PDB 2023 (5,300 models)	DeepRank-GNN	36	48	67	**100**	**100**
	GNN-DOVE	3	19	34	**100**	**100**
	dMaSIF	51	84	94	**100**	**100**
	PIsToN	**88**	**96**	**98**	**100**	**100**
	FireDock	0	3	11	**100**	**100**
	AP-PISA	0	1	5	**100**	**100**
	CP-PIE	76	92	96	**100**	**100**
	PyDock	0	0	3	**100**	**100**
	ZRANK2	0	11	23	**100**	**100**
	RosettaDock	3	17	32	**100**	**100**
	SIPPER	50	75	84	**100**	**100**
	HADDOCK	0	1	1	**100**	**100**

Bold values indicate the best value for that column

For almost all datasets, the DL-based methods outperformed the competing classical methods. For the CAPRI Score Refined dataset, BM4, and PDB-2023 datasets, PIsToN had the most number of top ranking performances for top 1, top 10, top 25, top 100, and top 200 predictions, with the exception of one value where HADDOCK outperformed other methods with 94% hits for top 25 prediction with only the BM4 dataset.

For the Easy and Difficult versions of the CAPRI Score v2022 datasets, DL-based methods demonstrated superior or equal performance compared to other methods for both difficult and easy complexes. PIsToN and dMaSIF achieved the best results for the top 1, with success rates of 17 and 51% for difficult and easy complexes, respectively. For the top 10, DeeRank-GNN and PIsToN had the highest success rates, with the former excelling in difficult complexes and the latter in easy targets. However, dMaSIF performed the best for both levels of difficulty in the top 25, the top 100, and the top 200. It achieved success rates of 46 and 89% for the top 25 in difficult and easy complexes, respectively. For the top 100 in easy and top 200 in difficult complexes, CP-PIE demonstrated the same performance as PIsToN, with success rates of 97% and 85%, respectively.

In order to more precisely examine the effectiveness of the methods in this metric and to better understand these percentages, Table 3 shows the number of times each method succeeded in finding a docking model with at least acceptable quality in the top 1 ranking. When considering only the top 1 ranking for each method, this count would reflect the number of complexes for which each method was successful in finding a correct docking model with at least acceptable quality as the top ranked solution.

The success rates of scoring functions on each dataset are depicted in Figure S1. The success rates of all scoring functions categorized by the CAPRI quality for the top 1, top 10, and top 100 rankings are reported in Table S4.

**Table 3:** The number of times a tool finds at least one docking model with acceptable quality in top 1

Method	CAPRI Score v2022 Difficult	CAPRI Score v2022 Easy	CAPRI Score Refined	BM4	BM5	Dockground	PDB-2023
# Complexes	44	39	13	61	176	231	53
DeepRank-GNN	6	14	3	4	**13**	22	19
GNN-DOVE	0	6	2	4	1	**41**	2
dMaSIF	6	**20**	2	9	6	21	27
PIsToN	**8**	16	**5**	**10**	9	15	**46**
FireDock	0	2	0	5	0	1	0
AP-PISA	0	6	0	5	0	1	0
CP-PIE	3	3	**5**	9	**13**	21	40
PyDock	0	2	0	3	0	0	0
ZRANK2	0	8	1	8	0	8	0
RosettaDock	1	9	0	8	2	7	2
SIPPER	1	10	1	9	9	27	26
HADDOCK	1	6	1	5	0	7	0

Bold values indicate the best value for that column

### Evaluation of AlphaFold3 docking models

AlphaFold3 [106], developed by DeepMind, is the latest version in the AlphaFold series. It represents a significant advancement in protein structure and complex predictions. AlphaFold3’s underlying model uses a diffusion architecture [107], which enables it to predict the correct coordinates of a molecule given their noisy atomic coordinates. AlphaFold3 has the capability to predict the structures of different kinds of biomolecules, including those involving proteins, nucleic acids, small molecules, and ions. AlphaFold3 evaluates docking models using various metrics. The predicted Local Distance Difference Test (pLDDT) [108] provides a confidence score for each atom, indicating the reliability of the predicted structure. The Predicted Aligned Error (PAE) estimates the positional error between two tokens in the predicted structure. The predicted Template Modeling (pTM) score [109] evaluates the overall structural accuracy, while the interface predicted Template Modeling (ipTM) score [109] assesses the accuracy of the predicted interactions between subunits. AlphaFold3 was released during the final stages of our study. The significance of this work encouraged us to include this section. The source code for AlphaFold3 is not publicly available yet, limiting direct access to it, although its server https://golgi.sandbox.google.com/about was accessible for our use.

To incorporate AlphaFold3 into our work and conduct experiments, we collected data from the eight datasets mentioned earlier with the exception of the MaSIF-test data set. MaSIF-test dataset contains only one correct and one incorrect docking model per complex, and due to the dataset’s large size relative to the other datasets, we randomly subsampled 10% of it. After consolidating all datasets, we eliminated duplicate complexes, resulting in a total of 280 complexes. For these complexes, we obtained their native structures from the Protein Data Bank [1].

Subsequently, we submitted jobs for these complexes to AlphaFold3’s server and received 5 docking models for each, resulting in a total of 1400 docking models. We then classified these models using CAPRI quality metrics, as detailed in the labeling data section. We differentiated the complexes for AlphaFold3 as those for which at least one correct docking model was generated regardless of its quality, and those for which no correct docking model was generated. The experiments in this section evaluate AlphaFold3 on the docking models generated with itself. The performance of others is reported from experiments conducted in Comparing Success Rate Measures section. Regarding the complexes for which AlphaFold3 was successful, we assessed its performance compared to other tools, and the findings are summarized in Table 4. For solutions of medium and high quality, AlphaFold3 demonstrated superior performance compared to other tools, achieving success rates of 17 and 39% for the top 1, respectively. Notably, dMaSIF outperformed other tools in the top 1 category for this level of quality. Additionally, PIsToN outperformed all of its competitors for the top 5 success rate across all three quality levels.

We conducted additional experiments for complexes for which AlphaFold3 could not predict a correct docking model. The results are summarized in Table 5. Since AlphaFold3 failed, we assigned zero to all of its columns. Among the top performers, GNN-DOVE had a 6% success rate for acceptable quality, PIsToN had a 13% success rate, and dMaSIF achieved a 32% success rate for high quality. Furthermore, dMaSIF outperformed other tools in both high and acceptable quality, with success rates of 37% and 20%, respectively, sharing the position for acceptable quality with PIsToN. For the top 5 medium-quality success, SIPPER, a classical method, achieved the best performance with a 30% success rate. We conclude that for complexes where AlphaFold3 failed to generate correct docking models, other docking methods may produce complexes of high quality. However, we confine our evaluations to scoring functions and not the docking methods.

**Table 4:** Success rates of all scoring functions with percentages of complexes for which AlphaFold3 generates at least one correct docking model

Model	Acceptable		Medium		High	
	Top1	Top5	Top1	Top5	Top1	Top5
AlphaFold3	6	11	**17**	24	**39**	41
AP_PISA	5	11	4	9	11	14
CP_PIE	5	24	14	31	22	42
DeepRank-GNN	7	24	15	28	20	32
FIREDOCK	4	10	3	12	5	17
GNN-DOVE	8	18	16	27	9	18
HADDOCK	4	15	2	15	0	16
PIsToN	10	**28**	**17**	**37**	33	**44**
PYDOCK_TOT	4	8	2	12	6	14
ROSETTADOCK	7	17	6	16	7	13
SIPPER	6	24	14	30	26	32
ZRANK2	6	12	6	15	12	14
dMaSIF	**11**	25	14	34	28	39

Bold values indicate the best value for that column

**Table 5:** Success rates of all scoring functions with percentages of complexes for which AlphaFold3 failed to generate a correct docking model

Model	Acceptable		Medium		High	
	Top1	Top5	Top1	top5	Top1	Top5
AlphaFold3	0	0	0	0	0	0
AP_PISA	2	9	6	13	13	18
CP_PIE	2	16	4	18	23	34
DeepRank-GNN	0	**20**	9	27	16	25
FIREDOCK	2	13	6	13	9	18
GNN-DOVE	**6**	13	11	27	6	23
HADDOCK	0	11	2	11	0	11
PIsToN	4	16	**13**	27	25	34
PYDOCK_TOT	2	9	4	13	13	18
ROSETTADOCK	0	16	6	16	7	18
SIPPER	2	16	6	**30**	27	30
ZRANK2	4	6	6	11	13	18
dMaSIF	4	**20**	6	27	**32**	**37**

Bold values indicate the best value for that column

### Comparing run times

**Fig. 3:**
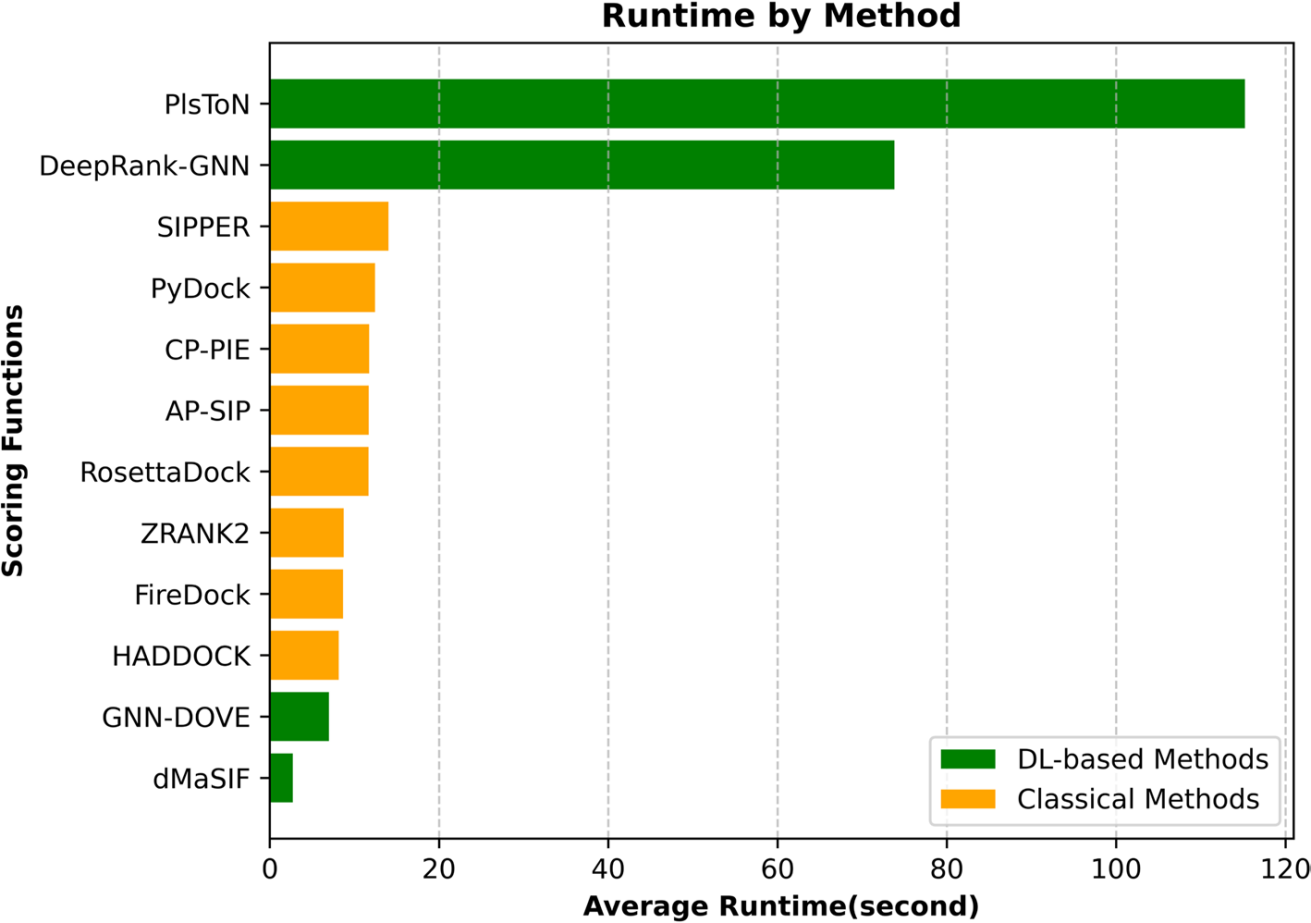
Average runtime of all twelve scoring functions on a dataset subsampled from the CAPRI Score Refined dataset

The running time of each scoring function was also evaluated, and the results are shown in Fig. 3. For this set of experiments, instead of running all the datasets, we subsampled the CAPRI Score Refined dataset to randomly select one correct and one acceptable docking model for each complex, resulting in a smaller dataset with 26 docking models for 13 complexes. The average run times from these experiments are thus averaged over complexes from different tools. These experiments were performed on a machine equipped with a GeForce GTX 1080 Ti graphics processing unit (GPU), 256 GB of RAM, and a 28-core Intel Xeon central processing unit (CPU) E5-2650.

As shown in Fig. 3, dMaSIF and GNN-DOVE, both DL-based methods, exhibit impressive efficiency, with average runtimes of 3 s and 7 s, respectively, surpassing the speed of all the classical methods. dMaSIF represents surface atoms as point clouds, calculating features on the fly without pre-computation, and this makes it faster than its competitors. We note that PIsToN and DeepRank-GNN have a preprocessing component included in the running time calculation. If preprocessing time is discounted, PIsToN requires 0.04 s, while DeepRank-GNN requires 6.3 s. Furthermore, since all classical tools were run on the CCharPPI server, their running times are approximate. The results show that DL-based methods are computationally more efficient than the classical methods.

### Assessing the generalizability of scoring functions

**Fig. 4:**
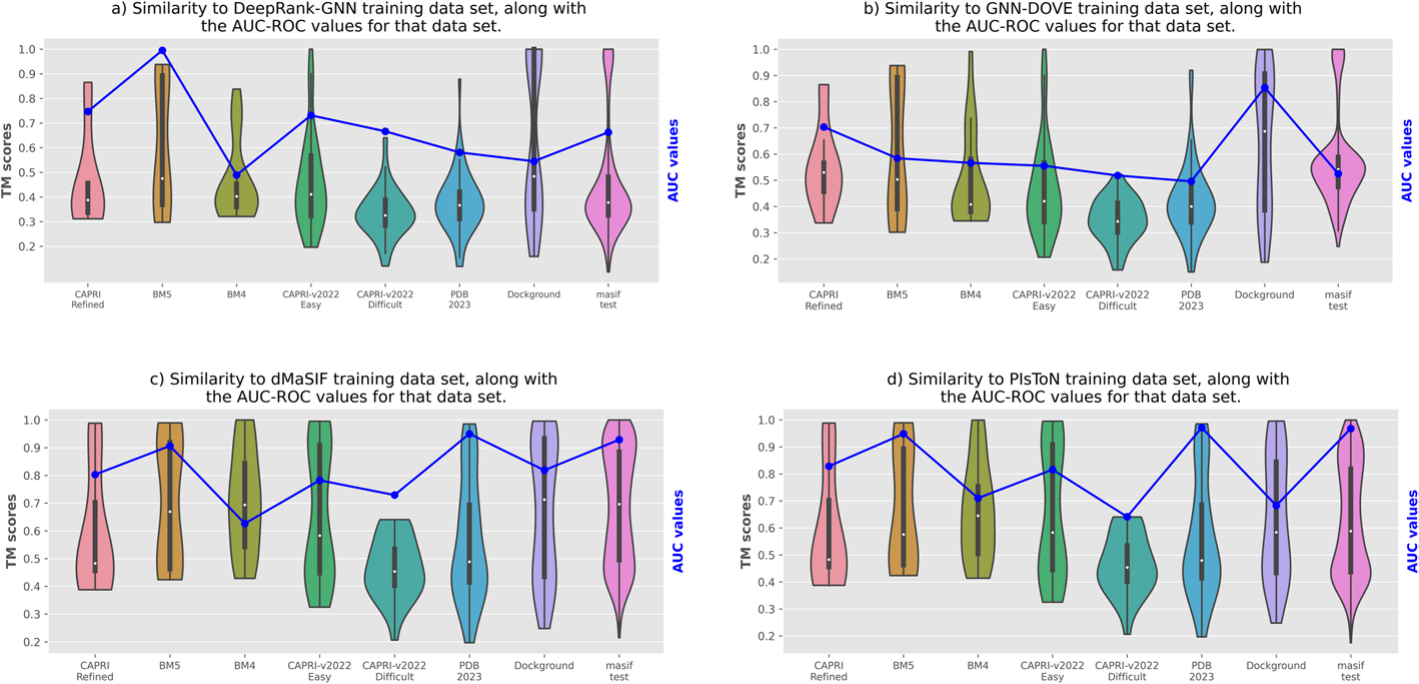
Distribution of the TM-scores between the training sets used by each method with the eight test datasets, along with the corresponding AUC values attained by that method for each test dataset. The limits of the black boxes inside the violin plots represent the 1st and 3rd quartiles, and the vertical lines (whiskers) represent the 1.5$\times$IQR interquartile range. The blue curve shows the AUC ROC values achieved by the method on the eight test datasets

To assess the ability of DL-based methods to generalize to out-of-distribution datasets, we utilized USalign version 2022092470 [110] to compute the Template Modeling Score (TM-score) for each pair of complexes in the test dataset and any complex in the training set separately for each method. The TM-score serves as a measure of *similarity* to the training set and ranges from 0 to 1, with 1 indicating an identical structure match. A TM-score of $\ge 0.5$ (or 0.45) signifies that the structures share the same global protein topology [110]. Therefore, a higher TM-score corresponds to greater similarity to the closest training complex and an indication of possible training bias in the testing. To understand this bias and to achieve a better evaluation and fair comparison, we plotted the distribution of TM-scores and the AUC ROC values for each method as shown in Figs. 4a–d. The AUC ROC values are indicated by the blue curve. It was superimposed on the violin plots. Note that a tool is more generalizable for a given data set if the TM scores are lower while the AUC ROC values are higher. The order in which the test datasets are shown is based on their size in terms of the number of complexes in the datasets (not the number of docking models), from smaller to larger (refer to Datasets section describing the data sets).

For the BM5 dataset, DeepRank-GNN performed the best in terms of having less similarity to the test dataset and achieving higher performance. Moreover, GNN-DOVE had a higher AUC value for the Dockground dataset. However, its training set shows the highest TM-score to the complexes of the Dockground dataset, which is not ideal. This means we cannot conclude that GNN-DOVE has the best generalization ability compared to others.

In the BM4 dataset, even though the training datasets of DeepRank-GNN and GNN-DOVE were the least similar to the test dataset, their performance was very poor. On the other hand, dMaSIF and PIsToN had training datasets with more similar complexes to BM4, yet they performed much better. In fact, PIsToN had the highest AUC value among all competitors, indicating its superior performance in this dataset.

For the CAPRI Refined, APRI scoreset v2022 (Difficult), and APRI scoreset v2022 (Easy) datasets, all methods had a TM-score of less than 0.5 and much wider around the median, except for GNN-DOVE for CAPRI Refined, which scored slightly higher than 0.5. This indicates that the training datasets used by all methods had little to no similarity to these datasets. On the other hand, dMaSIF and PIsToN demonstrated higher AUC values for these three datasets and outperformed DeepRank-GNN and GNN-DOVE. This confirmed that dMaSIF and PIsToN had very good generalization ability with the unseen data in these three datasets.

For PDB-2023 and MaSIF-test datasets, the dMaSIF training set showed the highest similarity, followed by PIsToN. However, their performance is significantly better than DeepRank-GNN and GNN-DOVE. The reason for this is that although dMaSIF and PIsToN were trained using similar complexes to these datasets, the diversity of this similarity is spread across the violin plot, indicating that it did not impact their generalizability.

We also note that the training data sets for DeepRank-GNN and GNN-DOVE were much smaller compared to those for dMaSIF and PIsToN. Additionally, the training data sets for dMaSIF is roughly the same size as that for PIsToN.

### Key factors affecting the performance of DL-based methods

To shed more light on the challenges faced by scoring functions in distinguishing between native and non-native docking models, we investigated the patterns of misclassifications of the tested docking models. We also queried what protein families may be involved in incorrect predictions. Additionally, the performance of DL-based methods is impacted by both the underlying training dataset, the relationship between the training and the test sets, and the architecture of the model.

To this end, we performed a series of experiments with the CAPRI score v2022 dataset. We selected this dataset because it is the largest and most diverse among all datasets, it includes both easy and difficult targets, and most importantly, none of the DL-based methods were trained on this dataset. The CAPRI score v2022 dataset contained 41,191 easy and 39,130 difficult models. Figures S2 and S4 and show a histogram of the number of methods that incorrectly classified each docking model from the easy and difficult cohorts. Of these, 652 easy and 512 difficult docking models were incorrectly classified by all 12 methods. We will refer to these two sets of docking models as the AllWrongSets. The protein family distributions of these sets of models can be found in Figures S10 and S11.

The next significant groups were the 1147 and 1520 models from the easy and difficult sets, respectively, for which only one method was successful. We will refer to these two sets of models as the AllButOneWrongSets, for which we determined how many docking models were correctly classified by each method (Figures S3 and S5). Next, we examined the distribution of protein families in the AllButOneWrongSets (Figures S12 and S13). In addition, we repeated the analysis with only the 4 DL-based methods (Figures S6 - S9). Finally, since DeepRank-GNN stood out as having been the method successful on the most number of models from the AllButOneWrongSets, we plotted the distribution of protein families for the set of models where it was the sole correct classifier (Figure S14 and S15). We used the TreeGrafter software [111] to identify the protein families to which these docking models belong. This software utilizes the trees in PANTHER database [112] to place a protein complex in the most suitable position on the phylogenetic tree.

Another factor contributing to the performance differences could be the docking tools and the resulting quality of the docking models used to train the models. Different methods employ different docking tools to generate docking models for their scoring tools. Therefore, it can be inferred that having high-quality docking models is a crucial step in developing an accurate and effective scoring function. Moreover, the features used to train a DL-based scoring function must be a significant factor as well. Important features play a crucial role in determining a model’s overall performance, as they directly influence the model’s ability to capture meaningful patterns and make accurate predictions. DeepRank-GNN is the only method out of the four DL-based methods in this study that included the type of amino acids and Position-Specific Scoring Matrix (PSSM) among its features. This might be one of the reasons for its good performance, while all other methods failed.

Similarity between the training dataset and the test dataset could be another reason for differing performance among the DL-based methods. Section Assessing the Generalizability of Scoring Functions investigates the similarity between this test dataset (CAPRI score v2022 dataset) and the training datasets of all DL-based methods.

## Discussion and conclusions

In this study, we conducted a comprehensive comparison between state-of-the-art DL-based and classical scoring functions. We used various popular datasets of different sizes. Among all classical methods, AP-PISA emerged as the top-performing method across all eight datasets. Unlike other classical methods that mainly consider potentials from the residue level, AP-PISA re-ranks refined docking models using a combination of atomic and residue potentials, which helps to identify near-native poses more accurately. On the other hand, AP-PISA relies solely on potential features, but incorporating energy terms can enhance performance by leveraging empirical and knowledge-based features. Overall, according to the AUC metric, CP-PIE was the second-best classical method and the best in ranking power. CP-PIE efficiently filters out unlikely docking poses before performing computationally intensive scoring calculations. It discards misdocked structures by measuring the overlap area between the proteins in each docking model. One conclusion that can be drawn is that in the refinement stage, deleting poses that are far away from the correct poses may be crucial for accurately scoring docking models.

In general, PIsToN and dMaSIF performed better than their DL-based competitors regarding both AUC and success rate metrics across all datasets. PIsToN utilized the strength of Vision Transformers [93] and contrastive training technique [113] to incorporate all chemical, spatial, and energy features, resulting in superior performance compared to other methods. However, PIsToN lagged behind its rival dMaSIF in terms of running time measurement due to its preprocessing step. PIsToN precomputes meshes for the surface of the two proteins involved in generating the complex to capture topological relationships and the geometry of their surfaces. While this precomputation enables it to be more accurate, it is a computationally intensive step. It may be beneficial for PIsToN to improve the preprocessing time by changing the surface representation to use non-Euclidean structural data like point clouds or graphs instead of meshes. In comparison, dMaSIF is very fast. It represents the surface atoms as point clouds, providing a flexible and simpler representation that can easily handle complex structures. For each atom, it only considers the atom type and its coordinates to calculate chemical features, avoiding the computation of physico-chemical features and significantly improving the runtime. However, dMaSIF estimates the chemical features based solely on the atom types and coordinates, failing to take into account the impact of other types of features in binding, such as energy terms, which can compromise accuracy.

Surprisingly, DeepRank-GNN, which is the third-best DL-based method in terms of an overall performance based on AUC values, had the most number of models in AllButOneWrongSets (Sect. Key Factors Affecting the Performance of DL-based Methods) for which it was the sole method that correctly distinguished near-native docking models from non-native ones. One factor contributing to its strong performance on the AllButOneWrongSets could be that DeepRank-GNN was trained on the BM5 [102] dataset, which was curated to have high diversity, containing all categories of protein complexes (antibody-antigen, enzyme-inhibitor, enzyme-substrate, etc.). The dMaSIF and PIsToN methods were not trained on BM5, but using a dataset extracted from the Protein Data Bank [1]; this training dataset was created using queries that took into account various chain sizes, chemical identifiers, and sequence similarity, but was not picked to guarantee diversity. While the use of Graph Neural Networks (GNN) in its architecture may be important, the fact that the other GNN-based method was not as successful as DeepRank-GNN on the AllButOneWrongSets suggests that the choice of GNN in the architecture may not be significant. More importantly, GNN-DOVE, was not trained on BM5, which again highlights the possibility of biases introduced by the training dataset. Furthermore, the results in Assessing the Generalizability of Scoring Functions section showed that the similarity between the CAPRI score v2022 test dataset and the training datasets of all DL-based methods is low and, therefore, could not be a reason for the performance differential seen in our study with the models in AllButOneWrongSets. Other factors, such as biases in the test dataset, could impact the performance of a DL method and needs to be addressed in future work.

Our study has demonstrated that DL-based techniques have proven to be significantly superior to classical methods for six out of eight datasets when measured by the AUC metric. Classical methods have limitations due to their inability to account for the 3D features and nonlinear relations between energy terms. This drawback affects their performance. Moreover, deep learning-based methods have consistently shown superior performance across all eight datasets when evaluated using the success rate metric. This is particularly important in fields such as drug discovery and protein engineering, where accurate identification of protein configurations can lead to fewer experiments and a decrease in experimental costs, thereby providing more reliability and cost efficiency in such applications.

As seen in Table 1, PIsToN and dMaSIF outperformed the other two DL-based models on six of the eight datasets. The superior performance of PIsToN and dMaSIF over the other two DL methods suggests that their models are better tuned to generalize to “out-of-distribution” datasets. The lower performance of GNN-DOVE and DeepRank-GNN may be attributed to their use of *K*-fold cross-validation, a technique that randomly partitions the dataset into training and test sets without paying attention to biases caused by similar samples falling on both sides of the partition. This might also explain why they only performed well on the datasets they were trained with. As mentioned earlier, DeepRank-GNN is the only method out of the four DL-based methods in this study that included the type of amino acids and Position-Specific Scoring Matrix (PSSM) among its features, which may have given it the ability to be the solitary method that was correct on the largest number of potential outlier models in AllButOneWrongSets.

In this study, we only evaluated rigid-body methods since most researchers have proposed scoring functions based on these methods. However, it might be worth exploring potential future directions to introduce scoring functions that also consider the flexibility of proteins during complex formation. Another potential path for future work could be to develop approaches for scoring multi-chain complexes and generating appropriate datasets to evaluate their performance.

## Key points

This paper provides a review of the literature on classical and deep learning-based scoring functions along with a fair comparison of the computational tools for scoring protein-protein docking. As computational biology and molecular modeling continue to advance through deep learning, it is crucial to evaluate both classical and DL approaches using various datasets to enable a consistent and fair comparison. Our study offers a comprehensive comparison of state-of-the-art methods using different well-known public datasets to help researchers better understand the progress in this field.

## Supplementary Information

Supplementary file 1.

## Data Availability

The datasets supporting the conclusions of this article are available in the CAPRI Score, https://scoreset.org/index.php?browse; CAPRI Score Refined and BM5, https://data.sbgrid.org/dataset/843/; 15 chosen complexes from BM5, https://zenodo.org/records/12681335; Dockground, http://dockground.compbio.ku.edu/downloads/unbound/decoy/decoys1.0.zip; BM4, http://zlab.umassmed.edu/ benchmark/; 19 chosen complexes from BM4, https://zenodo.org/records/12681335; PDB-2023, https://zenodo.org/record/7948337/files/PDB_2023.tar.gz; and MaSIF-test, https://zenodo.org/record/7948337/files/masif_test.tar.gz. The scripts that can be used to reproduce the results are now available on Zenodo at https://zenodo.org/records/12681335.

## References

[1] Berman HM, Westbrook J, Feng Z, Gilliland G, Bhat TN, Weissig H, Shindyalov IN, Bourne PE. The Protein Data Bank. Nucleic Acids Res. 2000;28(1):235–42. 10592235 10.1093/nar/28.1.235 PMC102472

[2] Huang S-Y. Search strategies and evaluation in protein-protein docking: principles, advances and challenges. Drug Discovery Today. 2014;19(8):1081–96. 24594385 10.1016/j.drudis.2014.02.005

[3] Vakser IA. Protein-protein docking: From interaction to interactome. Biophys J. 2014;107(8):1785–93. 25418159 10.1016/j.bpj.2014.08.033 PMC4213718

[4] Michael Gromiha M, Yugandhar K, and Sherlyn J. Protein-protein interactions: scoring schemes and binding affinity. Curr Opin Struct Biol, 2017; 44:31–8 10.1016/j.sbi.2016.10.016 27866112

[5] Pagadala NS, Syed K, Tuszynski J. Software for molecular docking: a review. Biophys Rev. 2017;9:91–102. 28510083 10.1007/s12551-016-0247-1 PMC5425816

[6] Chaudhary KK, Mishra N. A review on molecular docking: novel tool for drug discovery. Databases. 2016;3(4):1029.

[7] Huang S-Y. Exploring the potential of global protein-protein docking: an overview and critical assessment of current programs for automatic ab initio docking. Drug Discovery Today. 2015;20(8):969–77. 25801181 10.1016/j.drudis.2015.03.007

[8] Chen Y-C. Beware of docking! Trends Pharmacol Sci. 2015;36(2):78–95. 25543280 10.1016/j.tips.2014.12.001

[9] Hasani Horia Jalily, Barakat Khaled H. Protein-protein docking: Are we there yet? *Pharmaceutical Sciences: Breakthroughs in Research and Practice*, 2017;pages 1092–1114.

[10] Sunny S, Jayaraj PB Protein–protein docking: past, present, and future. *The protein journal*, 2022;1–26 10.1007/s10930-021-10031-8 34787783

[11] Stanzione F, Giangreco I, Cole JC. Use of molecular docking computational tools in drug discovery. Prog Med Chem. 2021;60:273–343. 34147204 10.1016/bs.pmch.2021.01.004

[12] Kaur T, Madgulkar A, Bhalekar M, Asgaonkar K. Molecular docking in formulation and development. Curr Drug Discov Technol. 2019;16(1):30–9. 29468973 10.2174/1570163815666180219112421

[13] Saikia S, Bordoloi M. Molecular docking: challenges, advances and its use in drug discovery perspective. Curr Drug Targets. 2019;20(5):501–21. 30360733 10.2174/1389450119666181022153016

[14] Li J, Fu A, Zhang L. An overview of scoring functions used for protein–ligand interactions in molecular docking. Interdiscip Sci Comput Life Sci. 2019;11:320–8. 10.1007/s12539-019-00327-w 30877639

[15] Sapundzhi F, Prodanova K, Lazarova M. Survey of the scoring functions for protein-ligand docking. In *AIP Conference Proceedings*, volume 2172. AIP Publishing 2019

[16] Vangone A, Oliva R, Cavallo L, Bonvin AMJJ (2017) Prediction of biomolecular complexes. *From protein structure to function with bioinformatics*, 265–292

[17] Huang S-Y, Grinter SZ, Zou X. Scoring functions and their evaluation methods for protein-ligand docking: recent advances and future directions. Phys Chem Chem Phys. 2010;12(40):12899–908. 20730182 10.1039/c0cp00151a PMC11103779

[18] Brian Pierce and Zhiping Weng. A combination of rescoring and refinement significantly improves protein docking performance. Proteins: Struct, Funct, Bioinf. 2008;72(1):270–9. 10.1002/prot.21920 PMC2696687 18214977

[19] Zimmermann MT, Leelananda SP, Kloczkowski A, Jernigan RL. Combining statistical potentials with dynamics-based entropies improves selection from protein decoys and docking poses. J Phys Chem B. 2012;116(23):6725–31. 22490366 10.1021/jp2120143

[20] Moreira IS, Martins JM, Coimbra JTS, Ramos MJ, Fernandes PA. A new scoring function for protein-protein docking that identifies native structures with unprecedented accuracy. Phys Chem Chem Phys. 2015;17(4):2378–87. 25490550 10.1039/c4cp04688a

[21] Zhang C, Liu S, Zhou Y. Docking prediction using biological information, ZDOCK sampling technique, and clustering guided by the DFIRE statistical energy function. Proteins: Struct, Funct, Bioinf. 2005;60(2):314–8. 10.1002/prot.20576 15981255

[22] Ohue M, Shimoda T, Suzuki S, Matsuzaki Y, Ishida T, Akiyama Y. MEGADOCK 4.0: an ultra-high-performance protein-protein docking software for heterogeneous supercomputers. Bioinformatics. 2014;30(22):3281–3. 25100686 10.1093/bioinformatics/btu532 PMC4221127

[23] Geng C, Jung Y, Renaud N, Honavar V, Bonvin AMJJ, Xue LC. iScore: a novel graph kernel-based function for scoring protein-protein docking models. Bioinformatics. 2020;36(1):112–21. 31199455 10.1093/bioinformatics/btz496 PMC6956772

[24] Moal IH, Bates PA. SwarmDock and the use of normal modes in protein-protein docking. Int J Mol Sci. 2010;11(10):3623–48. 21152290 10.3390/ijms11103623 PMC2996808

[25] Yang Y, Zhou Y. Ab initio folding of terminal segments with secondary structures reveals the fine difference between two closely related all-atom statistical energy functions. Protein Sci. 2008;17(7):1212–9. 18469178 10.1110/ps.033480.107 PMC2442011

[26] Yuedong Yang and Yaoqi Zhou. Specific interactions for ab initio folding of protein terminal regions with secondary structures. Proteins: Struct, Funct, Bioinf. 2008;72(2):793–803. 10.1002/prot.21968 18260109

[27] Brian Pierce and Zhiping Weng. ZRANK: reranking protein docking predictions with an optimized energy function. Proteins: Struct, Funct, Bioinf. 2007;67(4):1078–86. 10.1002/prot.21373 17373710

[28] Gray JJ, Moughon S, Wang C, Schueler-Furman O, Kuhlman B, Rohl CA, Baker D. Protein-protein docking with simultaneous optimization of rigid-body displacement and side-chain conformations. J Mol Biol. 2003;331(1):281–99. 12875852 10.1016/s0022-2836(03)00670-3

[29] Chinh Tran-To S, Nguyen T-D, Zheng J, Kwoh C-K. IFACEwat: the interfacial water-implemented re-ranking algorithm to improve the discrimination of near native structures for protein rigid docking. BMC Bioinf. 2014;15:1–15. 10.1186/1471-2105-15-S16-S9 PMC4290663 25521441

[30] Chowdhury R, Rasheed M, Keidel D, Moussalem M, Olson A, Sanner M, Bajaj C. Protein-protein docking with 2.0 and gb-rerank. PLoS ONE. 2013;8(3): e51307. 23483883 10.1371/journal.pone.0051307 PMC3590208

[31] Andrusier N, Nussinov R, Wolfson HJ. FireDock: fast interaction refinement in molecular docking. Proteins: Struct, Funct, Bioinf. 2007;69(1):139–59. 10.1002/prot.21495 17598144

[32] Dhawanjewar AS, Roy AA, Madhusudhan MS. A knowledge-based scoring function to assess quaternary associations of proteins. Bioinformatics. 2020;36(12):3739–48. 32246820 10.1093/bioinformatics/btaa207 PMC7425177

[33] Segura J, Marín-López MA, Jones PF, Oliva B, Fernandez-Fuentes N. VORFFIP-driven dock: , a fast and accurate protein docking strategy. PLoS ONE. 2015;10(3): e0118107. 25763838 10.1371/journal.pone.0118107 PMC4357426

[34] Krüger DM, Garzón JI, Chacón P, Gohlke H. knowledge-based potentials used as scoring and objective function in protein-protein docking. PLoS ONE. 2014;9(2): e89466. 24586799 10.1371/journal.pone.0089466 PMC3931789

[35] Malhotra S, Sankar K, Sowdhamini R. Structural interface parameters are discriminatory in recognising near-native poses of protein-protein interactions. PLoS ONE. 2014;9(2): e80255. 24498255 10.1371/journal.pone.0080255 PMC3912216

[36] Shruthi Viswanath DVS, Ravikant, and Ron Elber,. Improving ranking of models for protein complexes with side chain modeling and atomic potentials. Proteins Struct, Func, Bioinf. 2013;81(4):592–606. 10.1002/prot.24214 23180599

[37] Pons C, Talavera D, De La Cruz X, Orozco M, Fernandez-Recio J. Scoring by intermolecular pairwise propensities of exposed residues (SIPPER): a new efficient potential for protein- protein docking. J Chem Inf Model. 2011;51(2):370–7. 21214199 10.1021/ci100353e

[38] Vreven T, Hwang H, Weng Z. Integrating atom-based and residue-based scoring functions for protein-protein docking. Protein Sci. 2011;20(9):1576–86. 21739500 10.1002/pro.687 PMC3190152

[39] Liu S, Vakser IA. DECK: Distance and environment-dependent, coarse-grained, knowledge-based potentials for protein-protein docking. BMC Bioinf. 2011;12:1–7. 10.1186/1471-2105-12-280 PMC3145612 21745398

[40] DVS Ravikant and Ron Elber. Pie-efficient filters and coarse grained potentials for unbound protein-protein docking. Proteins: Struct, Funct, Bioinf. 2010;78(2):400–19. 10.1002/prot.22550 PMC2795038 19768784

[41] Lesk VI, Sternberg MJE. 3D-Garden: a system for modelling protein-protein complexes based on conformational refinement of ensembles generated with the marching cubes algorithm. Bioinformatics. 2008;24(9):1137–44. 18326508 10.1093/bioinformatics/btn093

[42] Mintseris J, Pierce B, Wiehe K, Anderson R, Chen R, Weng Z. Integrating statistical pair potentials into protein complex prediction. Proteins: Struct, Funct, Bioinf. 2007;69(3):511–20. 10.1002/prot.21502 17623839

[43] Jung Y, Geng C, Bonvin AMJJ, Xue LC, Honavar VG. MetaScore: a novel machine-learning-based approach to improve traditional scoring functions for scoring protein-protein docking conformations. Biomolecules. 2023;13(1):121. 36671507 10.3390/biom13010121 PMC9855734

[44] Stebliankin V, Shirali A, Baral P, Shi J, Chapagain P, Mathee K, Narasimhan G (2023) Evaluating protein binding interfaces with transformer networks. *Nat Mach Intell*, 1–12

[45] Réau M, Renaud N, Xue LC, Bonvin AMJJ. DeepRank-GNN: a graph neural network framework to learn patterns in protein-protein interfaces. Bioinformatics. 2023;39(1):btac759. 36420989 10.1093/bioinformatics/btac759 PMC9805592

[46] Guo L, He J, Lin P, Huang S-Y, Wang J. TRScore: a 3d RepVGG-based scoring method for ranking protein docking models. Bioinformatics. 2022;38(9):2444–51. 35199137 10.1093/bioinformatics/btac120

[47] Behbahani YM, Crouzet S, Laine E, Carbone A. Deep local analysis evaluates protein docking conformations with locally oriented cubes. Bioinformatics. 2022;38(19):4505–12. 35962985 10.1093/bioinformatics/btac551 PMC9525006

[48] Sverrisson F, Feydy J, Correia BE, Bronstein MM (2021) Fast end-to-end learning on protein surfaces. In *Proceedings of the IEEE/CVF Conference on Computer Vision and Pattern Recognition*, 15272–15281

[49] Renaud N, Geng C, Georgievska S, Ambrosetti F, Ridder L, Marzella DF, Réau MF, Bonvin AMJJ, Xue LC. DeepRank: a deep learning framework for data mining 3D protein-protein interfaces. Nat Commun. 2021;12(1):7068. 34862392 10.1038/s41467-021-27396-0 PMC8642403

[50] Wang X, Flannery ST, Kihara D. Protein docking model evaluation by graph neural networks. Front Mol Biosci. 2021;8: 647915. 34113650 10.3389/fmolb.2021.647915 PMC8185212

[51] Das S, Chakrabarti S. Classification and prediction of protein-protein interaction interface using machine learning algorithm. Sci Rep. 2021;11(1):1761. 33469042 10.1038/s41598-020-80900-2 PMC7815773

[52] Gainza P, Sverrisson F, Monti F, Emanuele Rodola D, Boscaini MMB, Correia BE. Deciphering interaction fingerprints from protein molecular surfaces using geometric deep learning. Nat Methods. 2020;17(2):184–92. 31819266 10.1038/s41592-019-0666-6

[53] Wang X, Terashi G, Christoffer CW, Zhu M, Kihara D. Protein docking model evaluation by 3D deep convolutional neural networks. Bioinformatics. 2020;36(7):2113–8. 31746961 10.1093/bioinformatics/btz870 PMC7141855

[54] Basu S, Wallner B. Finding correct protein-protein docking models using ProQDock. Bioinformatics. 2016;32(12):i262–70. 27307625 10.1093/bioinformatics/btw257 PMC4908341

[55] Akbal-Delibas B, Pomplun M, Haspel N. Accurate prediction of docked protein structure similarity. J Comput Biol. 2015;22(9):892–904. 26335807 10.1089/cmb.2015.0114 PMC4575526

[56] Cun-Xin WANG, Shan CHANG, Xin-Qi GONG, Feng YANG, Chun-Hua LI, Wei-Zu CHEN. Progress in the scoring functions of protein-protein docking. Acta Phys Chim Sin. 2012;28(4):751–8.

[57] Li CH, Ma XH, Shen LZ, Chang S, Chen WZ, Wang CX. Complex-type-dependent scoring functions in protein-protein docking. Biophys Chem. 2007;129(1):1–10. 17540496 10.1016/j.bpc.2007.04.014

[58] Li H, Sze K-H, Gang L, Ballester PJ. Machine-learning scoring functions for structure-based virtual screening. Wiley Interdiscip Rev: Comput Mol Sci. 2021;11(1): e1478. 10.1002/wcms.1225 PMC4832270 27110292

[59] Ballester PJ. Selecting machine-learning scoring functions for structure-based virtual screening. Drug Discov Today Technol. 2019;32:81–7. 33386098 10.1016/j.ddtec.2020.09.001

[60] Moal IH, Torchala M, Bates PA, Fernández-Recio J. The scoring of poses in protein-protein docking: current capabilities and future directions. BMC Bioinf. 2013;14(1):1–15. 10.1186/1471-2105-14-286 PMC3850738 24079540

[61] Minyi S, Qifan Yang YD, Feng G, Liu Z, Li Y, Wang R. Comparative assessment of scoring functions: the CASF-2016 update. J Chem Inf Model. 2018;59(2):895–913. 30481020 10.1021/acs.jcim.8b00545

[62] Lee M. Recent advances in deep learning for protein-protein interaction analysis: A comprehensive review. Molecules. 2023;28(13):5169. 37446831 10.3390/molecules28135169 PMC10343845

[63] Shen C, Ding J, Wang Z, Cao D, Ding X, Hou T. From machine learning to deep learning: advances in scoring functions for protein-ligand docking. Wiley Interdiscip Rev: Comput Mol Sci. 2020;10(1): e1429.

[64] Berend D, Xie X, Ma L, Zhou L, Liu Y, Xu C, Zhao J (2020) Cats are not fish: deep learning testing calls for out-of-distribution awareness. In *Proceedings of the 35th IEEE/ACM international conference on automated software engineering*, 1041–1052

[65] Ruping S (2001) Incremental learning with support vector machines. In *Proceedings 2001 IEEE international conference on data mining*, IEEE, 641–642.

[66] Cheng TM-K, Blundell TL, Fernandez-Recio J. pyDock: electrostatics and desolvation for effective scoring of rigid-body protein-protein docking. Proteins: Struct, Funct, Bioinf. 2007;68(2):503–15. 10.1002/prot.21419 17444519

[67] Chaudhury S, Lyskov S, Gray JJ. PyRosetta: a script-based interface for implementing molecular modeling algorithms using rosetta. Bioinformatics. 2010;26(5):689–91. 20061306 10.1093/bioinformatics/btq007 PMC2828115

[68] Dominguez C, Boelens R, Bonvin AMJJ. HADDOCK: a protein-protein docking approach based on biochemical or biophysical information. J Am Chem Soc. 2003;125(7):1731–7. 12580598 10.1021/ja026939x

[69] Teixeira João MC, Honorato Rodrigo V, Giulini M, Bonvin A, Alidoost S, Reys V, Jimenez B, Schulte D, Noort Charlotte van, Verhoeven S, Vreede B, Schott S, Tsai R (2024) haddocking/haddock3: v3.0.0-beta.5, Version 3.0.0-beta.5

[70] Moal IH, Jiménez-García B, Fernández-Recio J. CCharPPI web server: computational characterization of protein-protein interactions from structure. Bioinformatics. 2015;31(1):123–5. 25183488 10.1093/bioinformatics/btu594

[71] Yan Y, Tao H, He J, Huang S-Y. The HDOCK server for integrated protein-protein docking. Nat Protoc. 2020;15(5):1829–52. 32269383 10.1038/s41596-020-0312-x

[72] Kozakov D, Hall DR, Xia B, Porter KA, Padhorny D, Yueh C, Beglov D, Vajda S. The ClusPro web server for protein-protein docking. Nat Protoc. 2017;12(2):255–78. 28079879 10.1038/nprot.2016.169 PMC5540229

[73] Pierce BG, Wiehe K, Hwang H, Kim B-H, Vreven T, Weng Z. ZDOCK server: interactive docking prediction of protein-protein complexes and symmetric multimers. Bioinformatics. 2014;30(12):1771–3. 24532726 10.1093/bioinformatics/btu097 PMC4058926

[74] Schneidman-Duhovny D, Inbar Y, Nussinov R, Wolfson HJ (2005) PatchDock and SymmDock: servers for rigid and symmetric docking. *Nucleic acids research*, 33(suppl_2):W363–W367 10.1093/nar/gki481 PMC1160241 15980490

[75] Torchala M, Moal IH, Chaleil RAG, Fernandez-Recio J, Bates PA. SwarmDock: a server for flexible protein-protein docking. Bioinformatics. 2013;29(6):807–9. 23343604 10.1093/bioinformatics/btt038

[76] de Vries Sjoerd J, Schindler Christina EM, de Beauchêne I Chauvot, Zacharias M (2015) A web interface for easy flexible protein-protein docking with ATTRACT. *Biophysical journal*, 108(3):462–465 10.1016/j.bpj.2014.12.015 PMC4317529 25650913

[77] Tovchigrechko A, Vakser Ilya A (2006) GRAMM-X public web server for protein-protein docking. Nucleic acids research. 34(suppl_2):W310–4 10.1093/nar/gkl206 PMC1538913 16845016

[78] Jinchao Yu, Vavrusa M, Andreani J, Rey J, Tufféry P, Guerois R. InterEvDock: a docking server to predict the structure of protein-protein interactions using evolutionary information. Nucleic Acids Res. 2016;44(W1):W542–9. 27131368 10.1093/nar/gkw340 PMC4987904

[79] Macindoe G, Mavridis L, Venkatraman V, Devignes MD, Ritchie DW (2010) HexServer: an FFT-based protein docking server powered by graphics processors. Nucleic acids research. 38(suppl_2):W445–9 10.1093/nar/gkq311 PMC2896144 20444869

[80] Graves A (2012) Long short-term memory. *Supervised sequence labelling with recurrent neural networks*, 37–45

[81] Vaswani A, Shazeer N, Parmar N, Uszkoreit J, Jones L, Gomez AN, Kaiser Łukasz, Polosukhin I (2017) Attention is all you need. *Advances in neural information processing systems*, 30

[82] Krizhevsky A, Sutskever I, Hinton GE (2012) Imagenet classification with deep convolutional neural networks. *Advances in neural information processing systems*, 25

[83] Simonyan K, Zisserman A (2014) Very deep convolutional networks for large-scale image recognition. arXiv preprint arXiv:1409.1556

[84] ; Szegedy C, Liu W, Jia Y, Sermanet P, Reed S, Anguelov D, Erhan D, Vanhoucke V, Rabinovich A (2015) Going deeper with convolutions. In *Proceedings of the IEEE conference on computer vision and pattern recognition*, 1–9

[85] Scarselli F, Marco Gori A, Tsoi C, Hagenbuchner M, Monfardini G. The graph neural network model. IEEE Trans Neural Netw. 2008;20(1):61–80. 19068426 10.1109/TNN.2008.2005605

[86] Gilmer J, Schoenholz SS, Riley PF, Vinyals O, Dahl GE (2017) Neural message passing for quantum chemistry. In *International conference on machine learning*, pages 1263–1272. PMLR

[87] Wang Z, Yan W, Oates T (2017) Time series classification from scratch with deep neural networks: A strong baseline. In *2017 International joint conference on neural networks (IJCNN)*, pages 1578–1585. IEEE

[88] Dorffner G. Neural networks for time series processing. Neural Netw World. 1996;6(4):447–68.

[89] Shi J, Jain M, Narasimhan G (2022) Time Series Forecasting (TFS) using various deep learning models. arXiv preprint arXiv:2204.11115

[90] Shi J, Myana R, Stebliankin V, Shirali A, Narasimhan G (2023) Explainable parallel RCNN with novel feature representation for time series forecasting. arXiv preprint arXiv:2305.04876

[91] Velickovic P, Cucurull G, Casanova A, Romero A, Lio P, Bengio Y, et al. (2017) Graph Attention Networks Stat. 1050(20):10–48550

[92] Sazan M, Shamsuzzoha BM. EGRET: edge aggregated graph attention networks and transfer learning improve protein-protein interaction site prediction. Brief Bioinform. 2022;23(2):bbab578. 35106547 10.1093/bib/bbab578

[93] Dosovitskiy A, Beyer L, Kolesnikov A, Weissenborn D, Zhai X, Unterthiner T, Dehghani M, Minderer M, Heigold G, Gelly S, et al. (2020) An image is worth 16x16 words: Transformers for image recognition at scale. arXiv preprint arXiv:2010.11929

[94] ScoreSet. Homepage: https://scoreset.org, (2024). Accessed: 2024-06-01

[95] Lensink MF, Wodak SJ. Score_set: a CAPRI benchmark for scoring protein complexes. Proteins: Struct, Funct, Bioinf. 2014;82(11):3163–9. 10.1002/prot.24678 25179222

[96] Lensink MF, Nadzirin N, Velankar S, Wodak SJ. Modeling protein-protein, protein-peptide, and protein-oligosaccharide complexes: CAPRI 7th edition. Proteins: Struct, Funct, Bioinf. 2020;88(8):916–38. 10.1002/prot.25870 31886916

[97] Kryshtafovych A, Schwede T, Topf M, Fidelis K, Moult J. Critical assessment of methods of protein structure prediction (CASP)-Round XIV. Proteins: Struct, Funct, Bioinf. 2021;89(12):1607–17. 10.1002/prot.26237 PMC8726744 34533838

[98] Janin J. The targets of CAPRI rounds 13–19. Proteins: Struct, Funct, Bioinf. 2010;78(15):3067–72. 10.1002/prot.22774 20589643

[99] Janin J. The targets of CAPRI rounds 20–27. Proteins: Struct, Funct, Bioinf. 2013;81(12):2075–81. 10.1002/prot.24375 23900782

[100] Liu S, Gao Y, Vakser IA. Dockground protein-protein docking decoy set. Bioinformatics. 2008;24(22):2634–5. 18812365 10.1093/bioinformatics/btn497 PMC2579708

[101] Hwang H, Vreven T, Janin Joël, Weng Z (2010) Proteins: Structure, Function, and Bioinformatics. Protein-protein docking benchmark version 40. 78(15):3111–4 10.1002/prot.22830 PMC2958056 20806234

[102] Vreven T, Moal IH, Vangone A, Pierce BG, Kastritis PL, Torchala M, Chaleil R, Jiménez-García B, Bates PA, Fernandez-Recio J, et al. Updates to the integrated protein-protein interaction benchmarks: docking benchmark version 5 and affinity benchmark version 2. J Mol Biol. 2015;427(19):3031–41. 26231283 10.1016/j.jmb.2015.07.016 PMC4677049

[103] Renaud N, Geng C. PSSMGen: https://github.com/DeepRank/PSSMGen

[104] Collins Keeley W, Copeland Matthew M, Brysbaert Guillaume, Wodak Shoshana J, Bonvin Alexandre MJJ, Kundrotas Petras J, Vakser Ilya A, Lensink Marc F (2024) CAPRI-Q: The CAPRI resource evaluating the quality of predicted structures of protein complexes. *J Mol Biol*, 168540. 10.1016/j.jmb.2024.168540 PMC11458157 39237205

[105] Lensink MF, Méndez R, Wodak SJ. Docking and scoring protein complexes: CAPRI 3rd edition. Proteins: Struct, Funct, Bioinf. 2007;69(4):704–18. 10.1002/prot.21804 17918726

[106] Abramson J, Adler J, Dunger J, Evans R, Green T, Pritzel A, Ronneberger O, Willmore L, Ballard AJ, Bambrick J, et al. Accurate structure prediction of biomolecular interactions with AlphaFold 3. Nature. 10.1038/s41586-024-07487-w PMC11168924 38718835

[107] Karras T, Aittala M, Aila T, Laine S. Elucidating the design space of diffusion-based generative models. Adv Neural Inf Process Syst. 2022;35:26565–77.

[108] Mariani V, Biasini M, Barbato A, Schwede T. lDDT: a local superposition-free score for comparing protein structures and models using distance difference tests. Bioinformatics. 2013;29(21):2722–8. 23986568 10.1093/bioinformatics/btt473 PMC3799472

[109] Jinrui Xu and Yang Zhang. How significant is a protein structure similarity with TM-score= 0.5? Bioinformatics. 2010;26(7):889–95. 20164152 10.1093/bioinformatics/btq066 PMC2913670

[110] Zhang C, Shine M, Pyle AM, Zhang Y. Us-align: universal structure alignments of proteins, nucleic acids, and macromolecular complexes. Nat Methods. 2022;19(9):1109–15. 36038728 10.1038/s41592-022-01585-1

[111] Tang H, Finn RD, Thomas PD. TreeGrafter: phylogenetic tree-based annotation of proteins with Gene Ontology terms and other annotations. Bioinformatics. 2019;35(3):518–20. 30032202 10.1093/bioinformatics/bty625 PMC6361231

[112] Mi H, Huang X, Muruganujan A, Tang H, Mills C, Kang D, Thomas PD. PANTHER version 11: expanded annotation data from Gene Ontology and Reactome pathways, and data analysis tool enhancements. Nucleic Acids Res. 2017;45(D1):D183–9. 27899595 10.1093/nar/gkw1138 PMC5210595

[113] Khosla P, Teterwak P, Wang C, Sarna A, Tian Y, Isola P, Maschinot A, Liu C, Krishnan D. Supervised contrastive learning. Adv Neural Inf Process Syst. 2020;33:18661–73.

