# Supplementary material for: A comprehensive survey of scoring functions for protein docking models
Source: BMC Bioinformatics. 2025 Jan 22;26:25. doi: 10.1186/s12859-024-05991-4 (PMC11755896; doi:10.1186/s12859-024-05991-4)
Supplement: Supplementary file 1 [file 12859_2024_5991_MOESM1_ESM.pdf]

Appendix: Supplementary materials

Table S1: Docking tool and scoring function of each classical method

Method	Strategy/tool used for docking	Scoring function
FireDock [1]	PatchDock [2]	***Empirical-based:*** Integrates energy functions such as van der Waals, electrostatics, and atomic-contact energy. Includes adjustments of side-chain conformations and rigid-body positions.
PyDock [3]	ZDOCK and FTDOCK [4]	***Hybrid:*** Includes terms related to electrostatics, desolvation, and van der Waals energies, using a distance-dependent dielectric constant and weighted desolvation energies for the score.
RosettaDock [5]	Low-Resolution Monte Carlo Search & High-Resolution Refinement [5]	***Empirical-based:*** The Monte Carlo-based scoring function includes van der Waals forces, hydrogen bonding, and solvation effects. Novelty is in refining side-chain and backbone flexibility at high resolution.
ZRANK2 [6]	ZDOCK [7], ZRANK [8], and RosettaDock [5]	***Empirical-based:*** Uses a linear weighted combination of energy terms, including van der Waals energy, electrostatics, and desolvation to provide a fast but accurate scoring.
AP-PISA [9]	DOCK/PIE [10]	***Knowledge-based:*** A distance-dependent pairwise atomic potential, combined with a residue potential, is used to rescore the refined complexes.
CP-PIE [11]	An Efficient Filtering & Coarse-Grained Potentials [11]	***Knowledge-based:*** Computes the overlap and solvent-accessible surface areas and the number of residue contacts at the interface and uses it as a filter to eliminate non-native candidate docking models.
SIPPER [12]	FTDOCK [4]	***Knowledge-based:*** Assessed using a combination of residue-residue interface propensities for all residue pairs and the desolvation energy based on solvent-exposed area.
HADDOCK [13]	Ambiguous Interaction Restraints (AIRs) [13]	***Hybrid:*** Combines empirical interaction energy terms (van der Waals, electrostatics) with how well a model conforms to or deviates from the experimental data.

Table S2: Classification performance of scoring functions on seven datasets. The Columns correspond to the Area Under the Receiver Operating Characteristic curve (AUC ROC), Average Precision (AP), Balanced Accuracy (BA), and F1 scores, respectively. Bold values indicate the best value for each column.

Dataset	Method	AUC ROC	AP	BA	F1	Precision	Recall
**CAPRI Score v2022 (Difficult complexes)**	PIsToN	64.77	**3.02**	**63.05**	**66.16**	3.47	70.49
	dMaSIF	**71.84**	2.09	44.55	20.09	1.15	11.48
	DeepRank-GNN	69.54	2.56	50.67	20.72	**26.47**	1.44
	GNN-DOVE	55.13	2.62	53.12	17.73	6.73	19.09
	FireDock	59.47	2.70	58.49	15.43	2.81	77.83
	AP-PISA	58.08	2.48	54.57	15.50	2.99	34.50
	CP-PIE	59.91	1.94	41.21	13.11	1.61	45.93
	PyDock	62.68	2.70	57.75	16.02	3.21	48.96
	ZRANK2	63.83	2.75	57.40	16.72	3.71	35.89
	RosettaDock	55.33	2.31	52.13	40.59	2.39	55.18
	SIPPER	56.72	2.28	51.57	14.47	2.29	**93.62**
	HADDOCK	53.05	2.44	54.40	25.01	2.63	54.70
**CAPRI Score v2022 (Easy complexes)**	PIsToN	**80.15**	**46.24**	73.08	60.51	55.89	65.95
	dMaSIF	76.65	24.14	31.50	16.38	12.72	23.00
	DeepRank-GNN	74.75	27.67	50.15	8.14	**56.79**	4.10
	GNN-DOVE	58.30	30.35	53.62	18.07	53.23	10.88
	FireDock	75.06	40.31	69.50	55.64	45.15	72.48
	AP-PISA	79.41	45.89	**73.51**	**60.78**	53.82	69.80
	CP-PIE	78.14	27.53	49.45	1.38	2.25	0.71
	PyDock	75.17	40.56	69.94	56.12	45.03	74.44
	ZRANK2	72.32	40.99	70.15	56.38	46.04	72.71
	RosettaDock	70.13	40.74	68.40	54.31	49.46	60.21
	SIPPER	61.80	26.11	44.81	17.96	19.08	16.96
	HADDOCK	66.59	34.82	63.73	50.33	36.55	**80.80**
**CAPRI Score Refined**	PIsToN	81.25	24.86	73.24	42.21	30.37	69.16
	dMaSIF	78.53	12.33	49.14	21.89	12.33	97.69
	DeepRank-GNN	76.01	12.53	50.07	22.23	12.53	**99.81**
	GNN-DOVE	70.99	10.35	34.90	14.06	8.18	49.88
	FireDock	72.79	19.00	67.50	33.01	21.17	74.89
	AP-PISA	**84.87**	**30.18**	**75.36**	**49.29**	**39.80**	67.72
	CP-PIE	81.29	12.31	49.05	21.84	12.31	97.16
	PyDock	75.28	25.03	70.49	42.98	34.93	55.86
	ZRANK2	74.09	25.68	69.57	43.76	38.46	50.75
	RosettaDock	63.95	18.94	64.75	33.64	24.88	51.90
	SIPPER	53.03	13.89	55.25	23.62	14.97	55.95
	HADDOCK	70.34	21.59	66.06	37.91	32.29	45.88
**BM4**	PIsToN	**67.45**	**36.46**	**62.71**	**43.67**	**53.75**	36.77
	dMaSIF	62.50	26.27	49.64	41.44	26.27	98.16
	DeepRank-GNN	59.59	26.41	50.00	41.58	26.41	97.76
	GNN-DOVE	61.58	25.11	46.47	38.04	24.82	81.37
	FireDock	57.82	28.59	54.28	30.18	34.53	26.81
	AP-PISA	59.21	25.36	47.19	39.40	25.25	89.74
	CP-PIE	59.19	24.23	43.37	31.40	22.30	53.01
	PyDock	58.31	30.16	57.71	41.65	33.10	56.15
	ZRANK2	55.85	24.97	45.97	35.24	24.20	64.82
	RosettaDock	57.37	24.71	44.64	26.55	21.51	34.68
	SIPPER	59.87	24.50	42.28	18.21	16.80	19.88
	HADDOCK	56.10	26.46	50.13	41.76	26.46	**99.05**

Continued on next page

Table S3 – continued

Dataset	Method	AUC ROC	AP	BA	F1	Precision	Recall
**BM5**	PIsToN	91.84	23.64	74.90	45.14	38.16	55.24
	dMaSIF	87.89	4.39	27.24	5.86	3.11	51.05
	DeepRank-GNN	95.97	5.72	49.66	0.0	0.0	0.0
	GNN-DOVE	60.76	9.00	53.69	13.57	47.22	7.93
	FireDock	95.41	**43.12**	**85.00**	**63.96**	56.65	**74.43**
	AP-PISA	91.62	33.45	72.24	54.20	66.11	45.92
	CP-PIE	**97.16**	5.72	49.37	0.0	0.0	0.0
	PyDock	88.98	19.61	67.05	39.90	43.21	37.06
	ZRANK2	90.37	29.20	71.00	50.40	59.31	43.82
	RosettaDock	72.26	40.56	77.17	61.38	**67.99**	55.94
	SIPPER	87.70	4.86	18.09	2.67	1.43	20.05
	HADDOCK	90.85	29.91	78.87	51.94	44.44	62.47
**Dockground**	PIsToN	68.42	10.71	60.40	22.13	15.62	37.92
	dMaSIF	79.94	6.33	26.10	5.71	3.15	31.46
	DeepRank-GNN	59.76	7.75	50.21	3.93	9.16	25.00
	GNN-DOVE	**83.87**	6.24	23.61	5.53	3.03	31.46
	FireDock	56.08	8.50	54.82	15.69	8.82	70.83
	AP-PISA	79.19	**18.14**	**64.77**	**36.08**	**38.23**	34.17
	CP-PIE	72.26	6.50	38.67	10.65	5.81	63.75
	PyDock	77.17	13.71	64.10	28.99	23.01	39.17
	ZRANK2	64.18	10.78	58.76	22.35	18.93	27.29
	RosettaDock	61.62	9.41	57.17	18.50	12.80	33.33
	SIPPER	79.60	7.75	50.28	14.39	7.75	**99.17**
	HADDOCK	53.15	7.70	49.87	9.37	7.57	12.29
**PDB 2023**	PIsToN	**93.86**	**45.85**	**78.21**	**66.05**	**78.89**	56.80
	dMaSIF	91.83	1.85	23.46	2.11	1.08	42.40
	DeepRank-GNN	62.65	2.43	50.36	3.69	2.76	56.00
	GNN-DOVE	53.23	2.55	52.59	5.16	2.76	40.00
	FireDock	87.98	16.68	67.39	38.79	42.06	36.00
	AP-PISA	89.19	21.68	68.02	44.02	54.76	36.80
	CP-PIE	88.81	2.39	46.71	0.40	0.27	8.00
	PyDock	87.64	10.48	59.30	26.82	44.44	19.20
	ZRANK2	73.22	23.31	66.19	43.85	66.13	32.80
	RosettaDock	65.04	20.67	66.48	42.21	56.76	33.60
	SIPPER	76.25	2.39	49.52	4.65	2.39	**93.60**
	HADDOCK	84.36	26.09	67.79	47.12	68.18	36.00
**MaSIF test**	PIsToN	**93.55**	**81.08**	**85.43**	**85.97**	87.72	82.40
	dMaSIF	89.90	45.55	19.60	13.52	14.63	12.57
	DeepRank-GNN	69.24	47.92	45.64	62.05	47.66	88.90
	GNN-DOVE	55.71	50.85	51.63	38.76	52.81	30.62
	FireDock	77.82	48.33	46.52	62.79	48.15	90.24
	AP-PISA	76.43	49.13	48.22	64.32	49.07	**93.34**
	CP-PIE	76.98	63.55	70.56	74.29	65.94	85.06
	PyDock	74.73	48.53	46.97	63.40	48.40	91.86
	ZRANK2	74.66	49.06	48.08	64.11	48.98	92.75
	RosettaDock	71.17	49.13	48.22	63.99	49.05	92.01
	SIPPER	66.17	56.01	60.43	66.63	57.61	78.99
	HADDOCK	81.85	73.94	76.55	71.78	**90.16**	59.62

Bold values indicate the best value for each column.

Table S4: Success rates of all twelve scoring functions on all datasets. The top four methods are DL-based, and the rest are classical methods

**Dataset**	**Method**	**acceptable**			**medium**			**high**		
		**top1**	**top10**	**top100**	**top1**	**top10**	**top100**	**top1**	**top10**	**top100**
**CAPRI Score v2022 (Difficult)**	DeepRank-GNN	7	**32**	65	0	8	30	0	0	0
	GNN-DOVE	0	7	35	0	0	17	0	0	0
	dMaSIF	10	28	**75**	**3**	**10**	32	0	0	0
	PIsToN	**17**	28	67	0	3	**35**	0	0	0
	FireDock	0	0	25	0	0	10	0	0	0
	AP_PISA	0	10	39	0	3	7	0	0	0
	CP_PIE	7	25	53	0	0	28	0	0	0
	PyDock	0	7	35	0	0	7	0	0	0
	ZRANK2	0	14	46	0	0	25	0	0	0
	RosettaDock	3	10	46	0	0	28	0	0	0
	SIPPER	0	14	50	**3**	7	25	0	0	0
	HADDOCK	3	14	46	0	0	17	0	0	0
**CAPRI Score v2022 (Easy)**	DeepRank-GNN	15	43	74	20	64	82	0	16	30
	GNN-DOVE	2	25	66	12	35	58	0	5	15
	dMaSIF	**28**	41	79	15	48	**87**	**7**	**23**	**38**
	PIsToN	17	**51**	**84**	20	**66**	**87**	2	20	30
	FireDock	2	15	46	2	5	25	0	5	12
	AP_PISA	7	17	66	7	17	46	0	2	10
	CP_PIE	5	35	**84**	**25**	48	**87**	5	12	28
	PyDock	2	10	46	2	5	43	0	5	7
	ZRANK2	10	25	74	10	30	69	0	2	10
	RosettaDock	12	30	71	10	25	69	0	2	15
	SIPPER	10	38	82	15	43	79	0	7	30
	HADDOCK	7	41	69	5	30	69	2	10	17
**CAPRI Scores Refined**	DeepRank-GNN	7	38	61	15	38	53	0	7	15
	GNN-DOVE	0	38	**76**	7	23	53	0	**15**	23
	dMaSIF	**15**	30	**76**	0	15	**69**	0	**15**	**38**
	PIsToN	7	**46**	61	**30**	**46**	53	0	7	23
	FireDock	0	0	23	0	0	0	0	0	0
	AP_PISA	0	0	15	0	0	7	0	0	0
	CP_PIE	7	30	61	7	23	61	**7**	7	30
	PyDock	0	0	0	0	0	0	0	0	0
	ZRANK2	0	0	46	0	7	23	0	7	7
	RosettaDock	0	0	61	0	7	30	0	7	7
	SIPPER	7	38	53	0	15	61	0	0	23
	HADDOCK	0	0	53	0	7	38	0	7	7
**BM4**	DeepRank-GNN	0	15	36	0	21	36	**5**	**21**	36
	GNN-DOVE	0	15	**47**	0	21	42	0	10	42
	dMaSIF	**5**	5	31	0	15	31	**5**	10	31
	PIsToN	**5**	26	42	5	**26**	42	**5**	**21**	42
	FireDock	**5**	15	31	0	10	31	0	10	31
	AP_PISA	0	**31**	36	0	**26**	36	0	15	26
	CP_PIE	0	15	**47**	**15**	21	42	**5**	15	42
	PyDock	0	15	31	0	15	31	0	5	26
	ZRANK2	0	5	**47**	0	21	**47**	0	10	**47**
	RosettaDock	0	10	**47**	0	10	**47**	0	5	**47**
	SIPPER	0	15	31	0	21	31	0	10	26

Continued on next page

Table S5 – continued

Dataset	Method	acceptable			medium			high		
		**top1**	**top10**	**top100**	**top1**	**top10**	**top100**	**top1**	**top10**	**top100**
	HADDOCK	**5**	15	**47**	5	15	**47**	0	10	**47**
**BM5**	DeepRank-GNN	**13**	46	**80**	**40**	**66**	**66**	40	80	**100**
	GNN-DOVE	0	6	33	0	13	20	6	6	20
	dMaSIF	0	26	**80**	13	33	**66**	26	73	**100**
	PIsToN	6	40	**80**	**40**	60	**66**	20	73	**100**
	FireDock	0	0	6	0	0	0	0	0	0
	AP_PISA	0	6	13	0	0	0	0	0	0
	CP_PIE	6	**53**	**80**	**40**	60	**66**	**46**	**86**	**100**
	PyDock	0	0	6	0	0	0	0	0	0
	ZRANK2	0	0	6	0	0	0	0	0	0
	RosettaDock	**13**	46	66	0	6	20	0	0	0
	SIPPER	0	40	**80**	20	60	**66**	40	73	93
	HADDOCK	0	0	6	0	0	0	0	0	0
**Dockground**	DeepRank-GNN	1	10	26	35	59	**98**	1	3	**17**
	GNN-DOVE	**12**	**22**	26	**57**	**82**	**98**	1	12	**17**
	dMaSIF	5	15	26	29	75	**98**	1	12	**17**
	PIsToN	0	1	**28**	24	71	**98**	1	8	**17**
	FireDock	0	7	26	1	14	96	0	0	15
	AP_PISA	0	3	26	1	17	92	0	0	12
	CP_PIE	3	10	**28**	29	64	**98**	3	**14**	**17**
	PyDock	0	1	26	0	10	89	0	0	12
	ZRANK2	1	1	22	12	42	**98**	0	0	14
	RosettaDock	1	3	21	10	47	94	0	0	15
	SIPPER	3	14	26	35	77	**98**	**8**	12	**17**
	HADDOCK	3	5	26	10	56	**98**	0	1	14
**PDB 2023**	DeepRank-GNN	1	11	**48**	3	5	**30**	30	48	**96**
	GNN-DOVE	3	15	**48**	0	0	**30**	0	5	**96**
	dMaSIF	1	44	**48**	3	26	**30**	46	80	**96**
	PIsToN	1	44	**48**	**9**	**30**	**30**	**76**	**92**	**96**
	FireDock	0	3	**48**	0	0	**30**	0	0	**96**
	AP_PISA	0	1	**48**	0	0	**30**	0	0	**96**
	CP_PIE	0	38	**48**	1	19	**30**	75	90	**96**
	PyDock	0	0	**48**	0	0	**30**	0	0	**96**
	ZRANK2	0	7	**48**	0	3	**30**	0	0	**96**
	RosettaDock	3	11	**48**	0	5	**30**	0	0	**96**
	SIPPER	3	38	**48**	7	17	**30**	38	65	**96**
	HADDOCK	0	1	**48**	0	0	**30**	0	0	**96**

Bold values indicate the best value for each column.

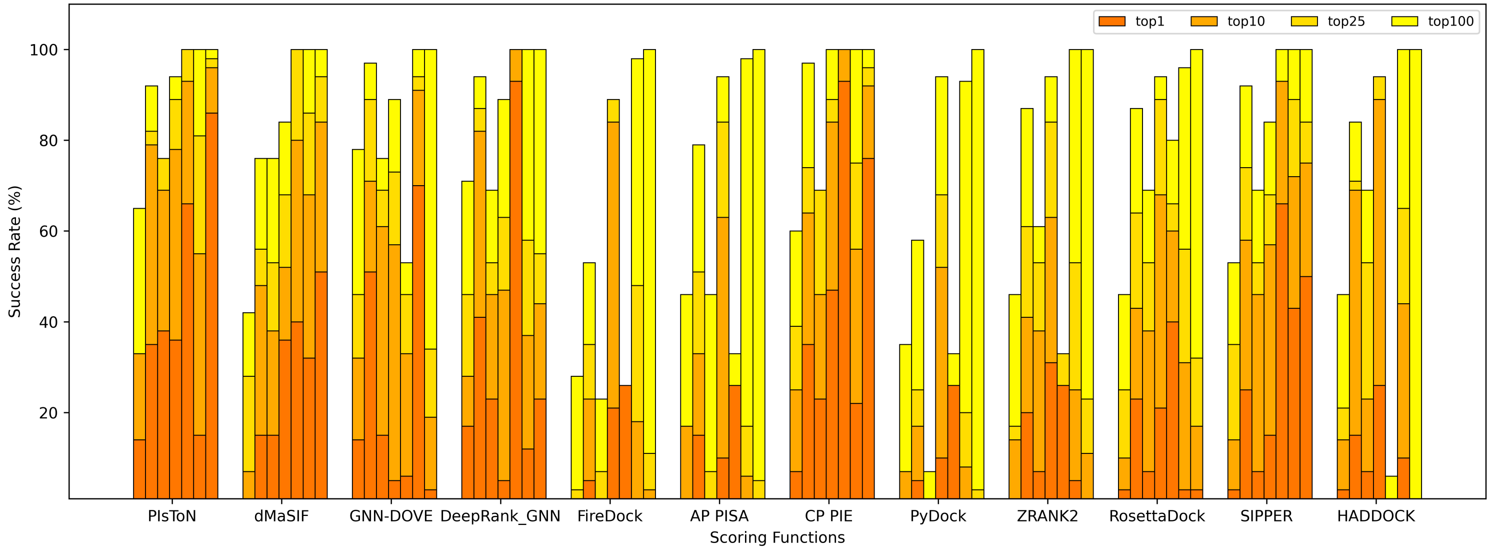

Figure S1: The success rates of scoring functions. For each method, the columns correspond to datasets (from left to right: CAPRI Score v2022 (Difficult), CAPRI Score v2022 (Easy), CAPRI Score Refined, BM4, BM5, Dockground, and PDB-2023). The colors correspond to the top 1, top 10, top 25, and top 100.

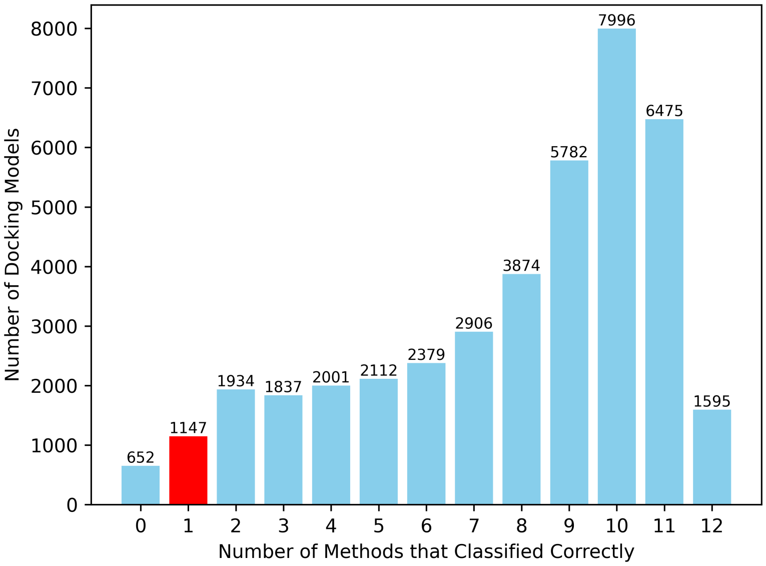

Figure S2: Correct classifications by methods for the “AllButOneWrongSets” set (CAPRI Score v2022 - easy targets)

Figure S2 illustrates that 1147 easy docking models were correctly classified by only one method (highlighted in red) in the “AllButOneWrongSets” set. For further clarification, Figure S3 shows how many docking models were classified correctly with each method.

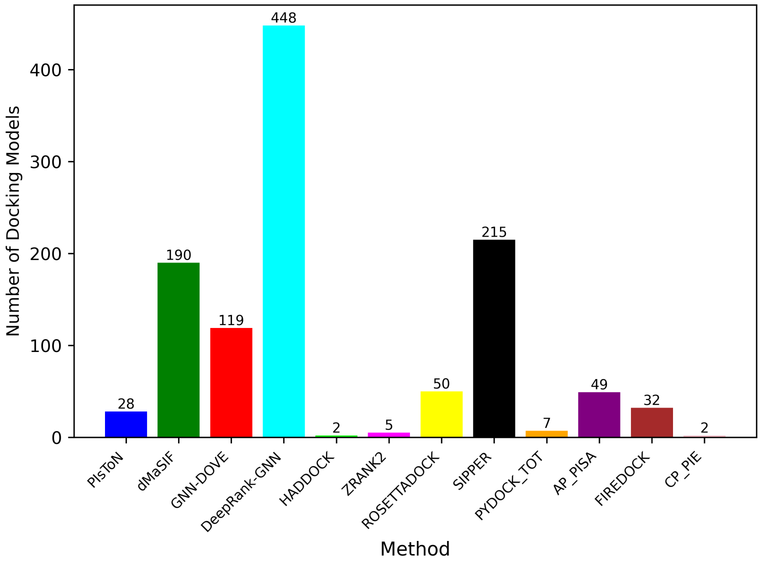

Figure S3: Distribution of the number of docking models correctly classified by only one method in the “AllButOneWrongSets” set (CAPRI Score v2022 - easy targets)

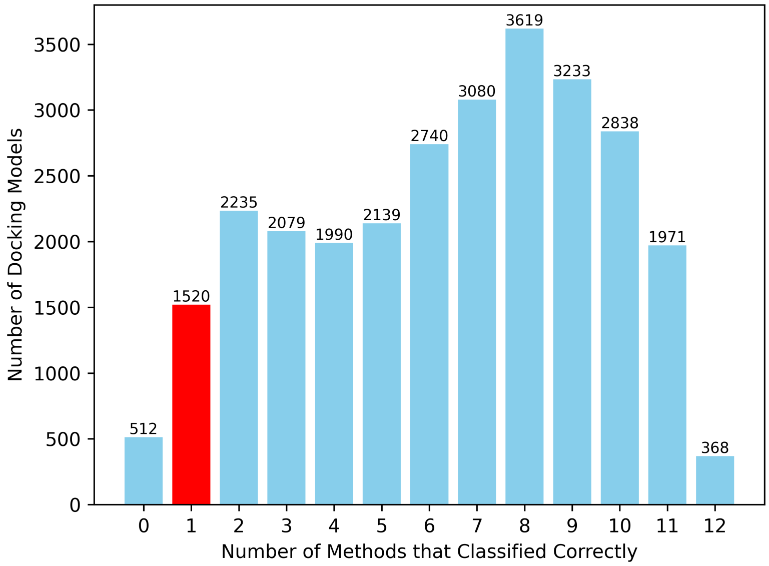

Figure S4: Correct classifications by methods for the "AllButOneWrongSets" set (CAPRI Score v2022 - difficult targets)

Figure S4 illustrates that 1520 difficult docking models were correctly classified by only one method (highlighted in red) in the "AllButOneWrongSets" set. For further clarification, Figure S5 shows how many docking models were classified correctly with each method.

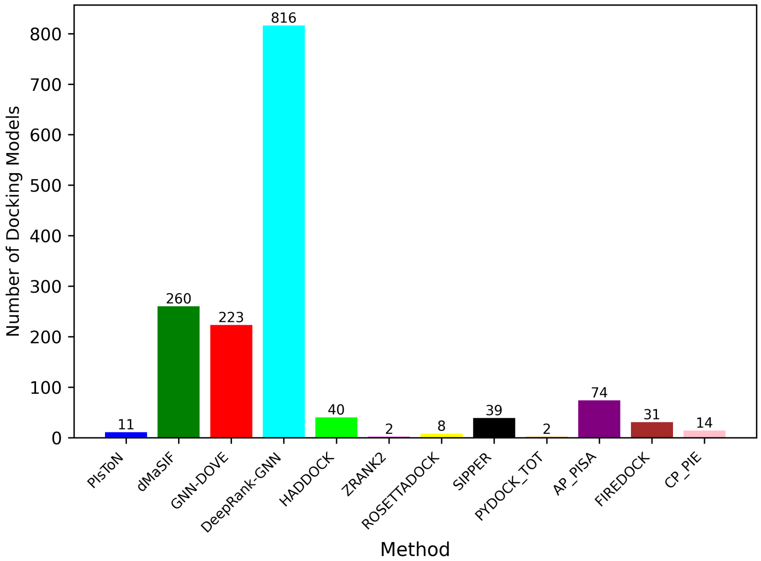

Figure S5: Distribution of the number of docking models correctly classified by only one method in the "AllButOneWrongSets" set (CAPRI Score v2022 - difficult targets)

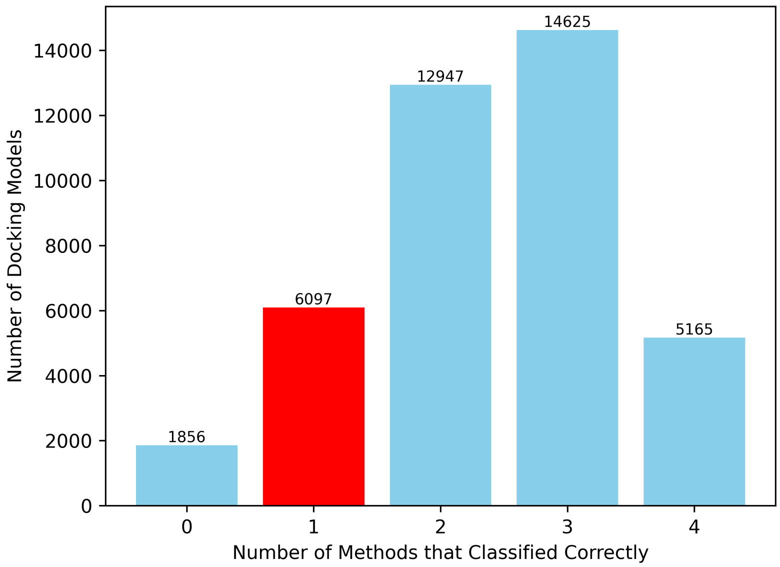

Figure S6: Correct classifications by DL-based methods for the "AllButOneWrongSets" set (CAPRI Score v2022 - easy targets)

Figure S6 illustrates that 6097 easy docking models were correctly classified by only one DL-based method (highlighted in red) in the "AllButOneWrongSets" set. For further clarification, Figure S7 shows how many docking models were classified correctly with each DL-based method.

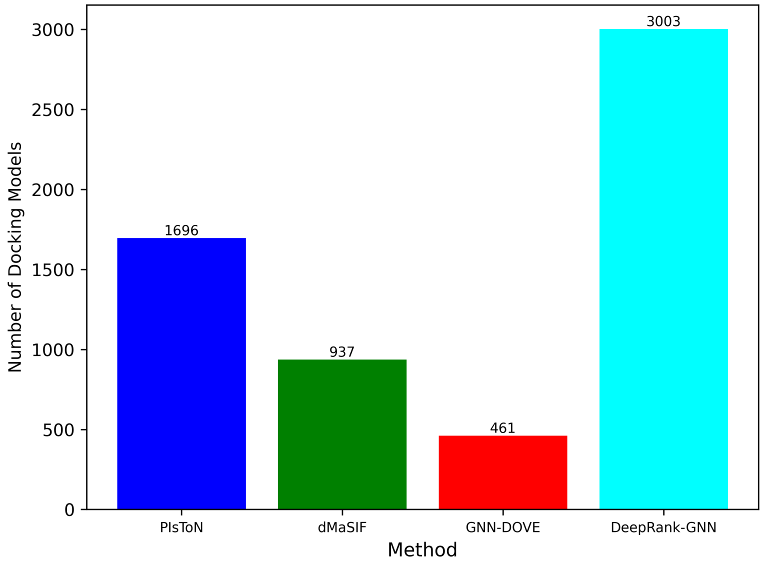

Figure S7: Distribution of the number of docking models correctly classified by only one DL-based method in the "AllButOneWrongSets" set (CAPRI Score v2022 - easy targets)

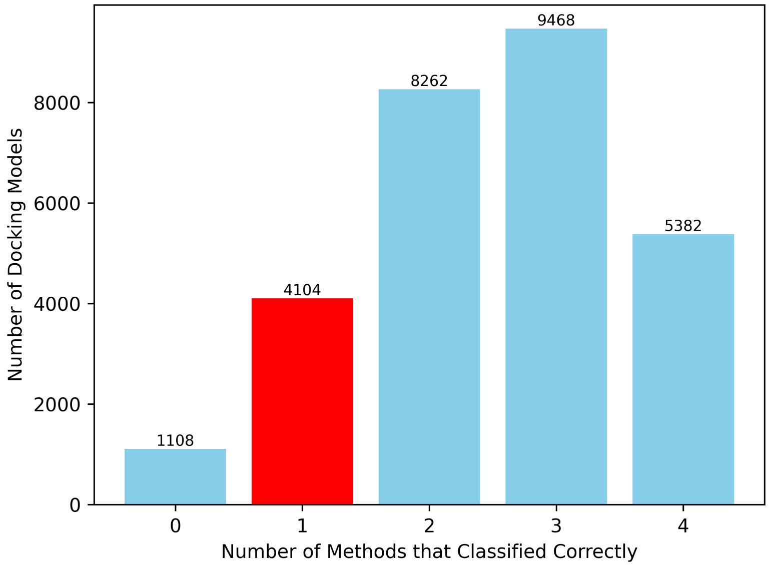

Figure S8: Correct classifications by DL-based methods for the “AllButOneWrongSets” set (CAPRI Score v2022 - difficult targets)

Figure S8 illustrates that 4104 difficult docking models were correctly classified by only one DL-based method (highlighted in red) in the “AllButOneWrongSets” set. For further clarification, Figure S9 shows how many docking models were classified correctly with each DL-based method.

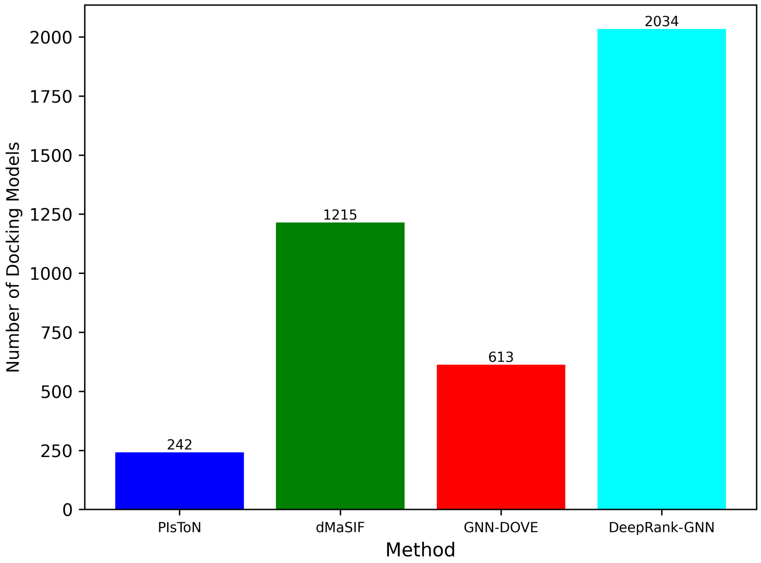

Figure S9: Distribution of the number of docking models correctly classified by only one DL-based method in the “AllButOneWrongSets” set (CAPRI Score v2022 - difficult targets)

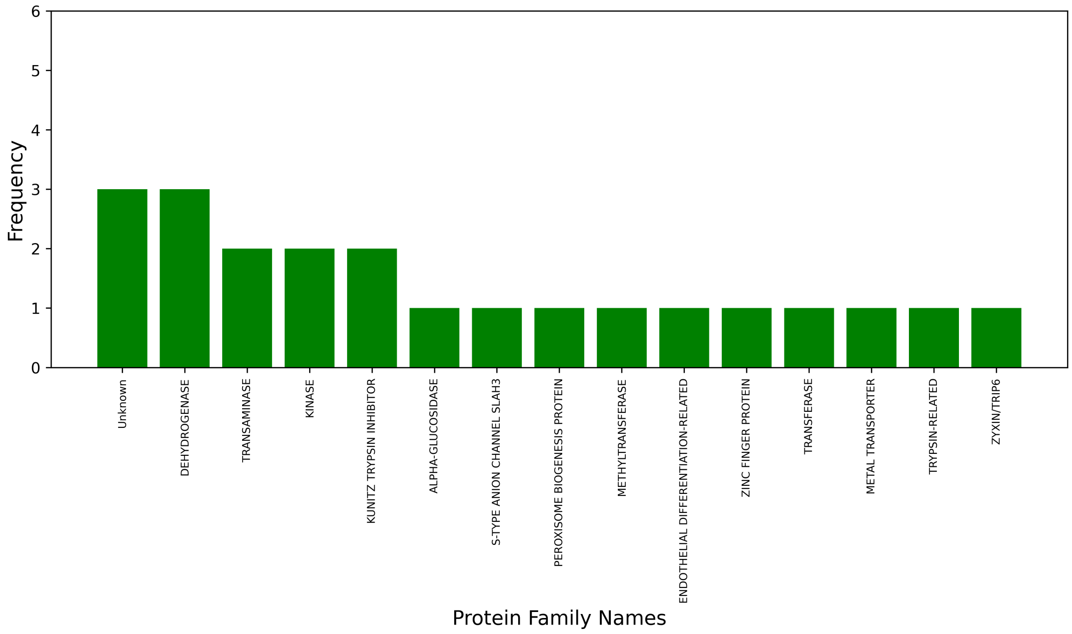

Figure S10: Distribution of protein families of the “AllWrongSets” set (CAPRI Score v2022 - easy targets)

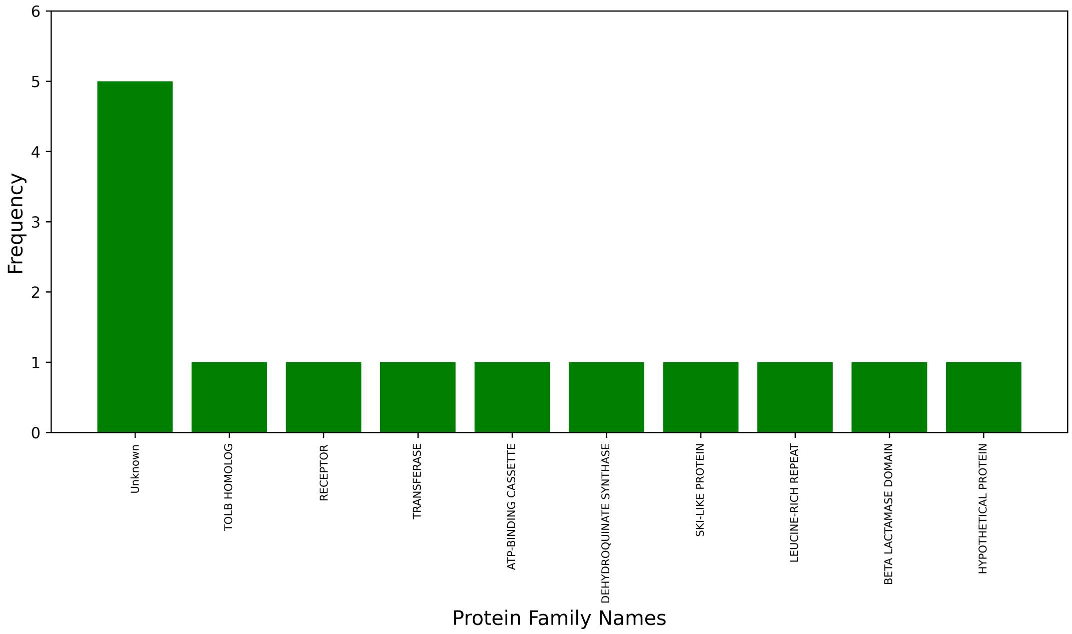

Figure S11: Distribution of protein families of the “AllWrongSets” set (CAPRI Score v2022 - difficult targets)

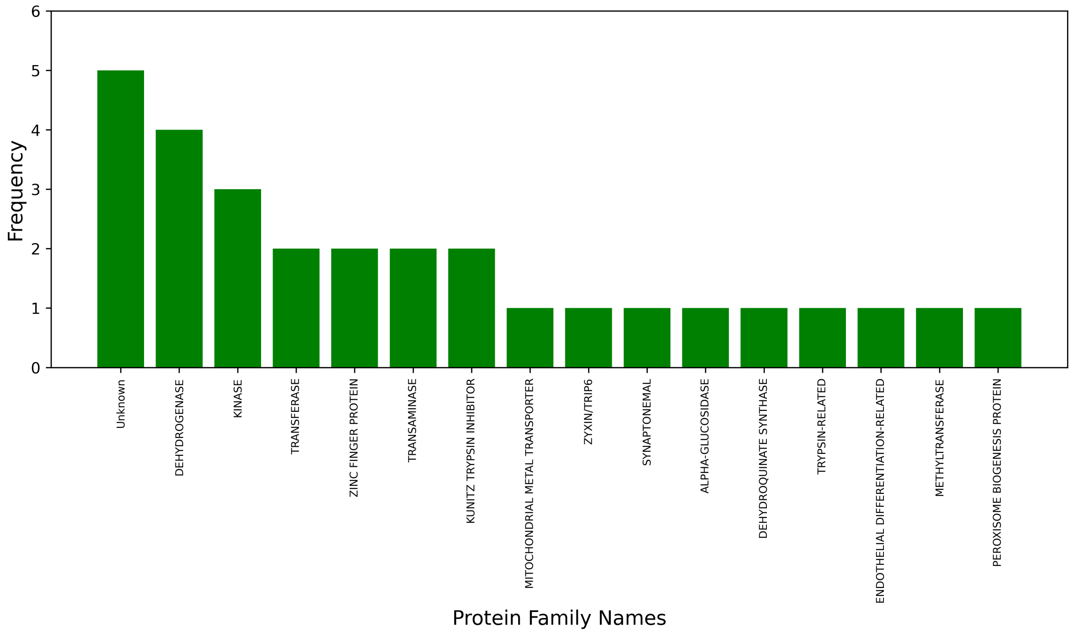

Figure S12: Distribution of protein families of the “AllButOneWrongSets” set (CAPRI Score v2022 - easy targets)

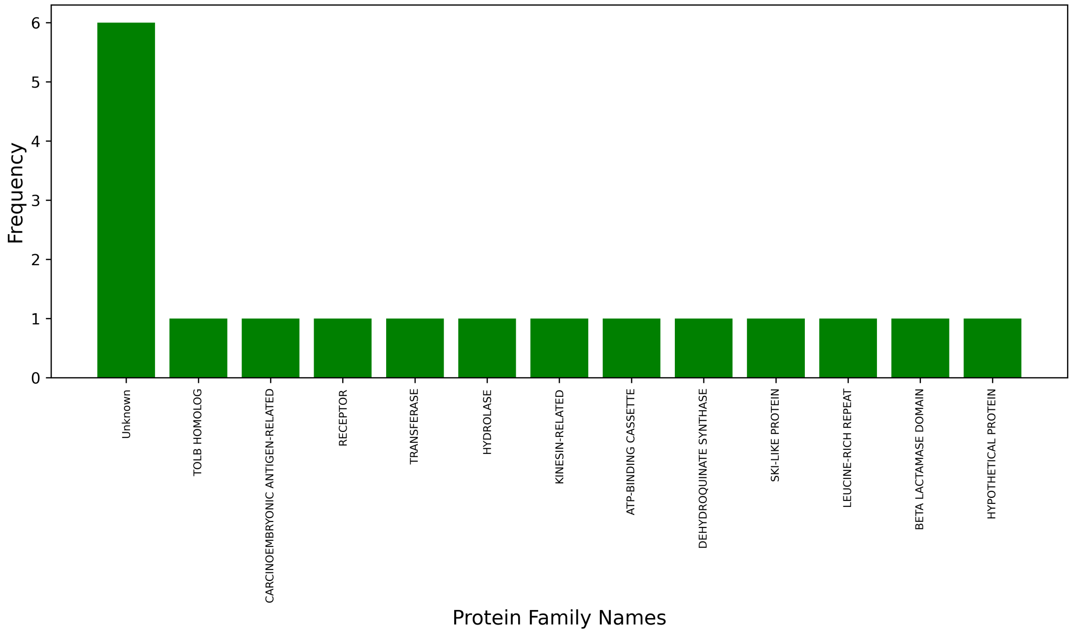

Figure S13: Distribution of protein families of the “AllButOneWrongSets” set (CAPRI Score v2022 - difficult targets)

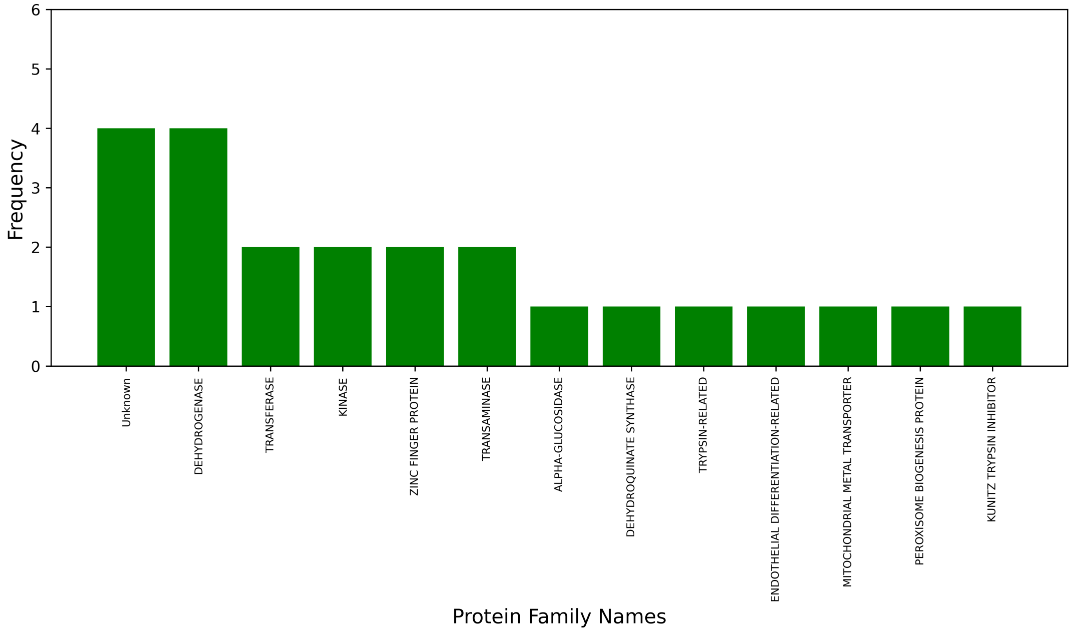

Figure S14: Distribution of protein families for which DeepRank-GNN was the only successful method in the “AllButOneWrongSets” set
(CAPRI Score v2022 - easy targets)

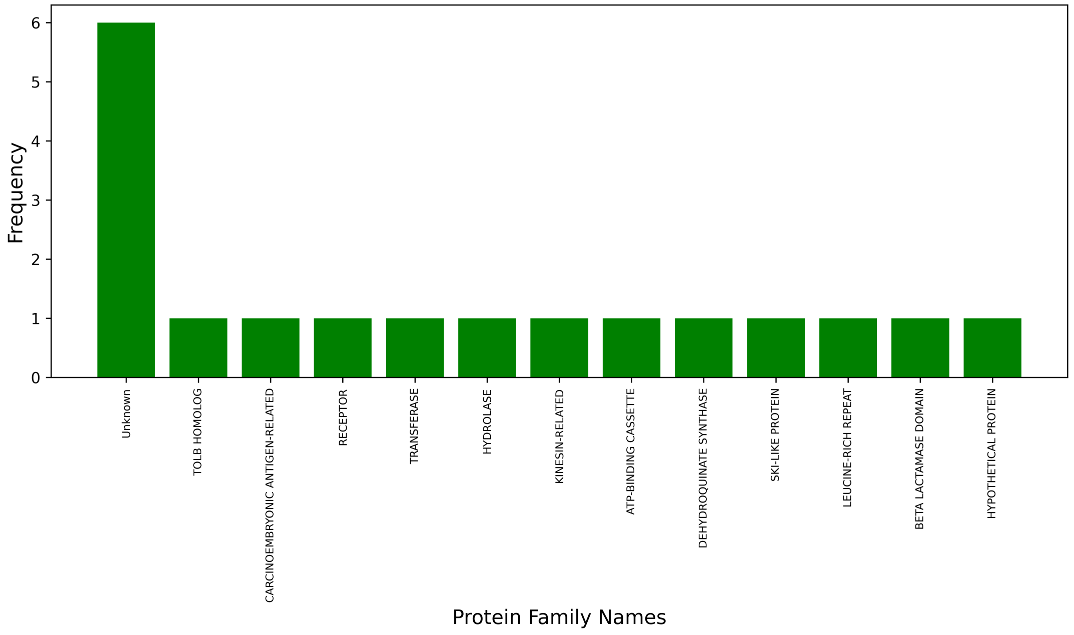

Figure S15: Distribution of protein families for which DeepRank-GNN was the only successful method in the “AllButOneWrongSets” set
(CAPRI Score v2022 - difficult targets)

References

[1] Nelly Andrusier, Ruth Nussinov, and Haim J Wolfson. FireDock: fast interaction refinement in molecular docking. *Proteins: Structure, Function, and Bioinformatics*, 69(1):139–159, 2007.

[2] Dina Schneidman-Duhovny, Yuval Inbar, Ruth Nussinov, and Haim J Wolfson. PatchDock and SymmDock: servers for rigid and symmetric docking. *Nucleic acids research*, 33(suppl_2):W363–W367, 2005.

[3] Tammy Man-Kuang Cheng, Tom L Blundell, and Juan Fernandez-Recio. pyDock: electrostatics and desolvation for effective scoring of rigid-body protein–protein docking. *Proteins: Structure, Function, and Bioinformatics*, 68(2):503–515, 2007.

[4] Henry A Gabb, Richard M Jackson, and Michael JE Sternberg. Modelling protein docking using shape complementarity, electrostatics and biochemical information. *Journal of molecular biology*, 272(1):106–120, 1997.

[5] Jeffrey J Gray, Stewart Moughon, Chu Wang, Ora Schueler-Furman, Brian Kuhlman, Carol A Rohl, and David Baker. Protein–protein docking with simultaneous optimization of rigid-body displacement and side-chain conformations. *Journal of molecular biology*, 331(1):281–299, 2003.

[6] Brian Pierce and Zhiping Weng. A combination of rescoring and refinement significantly improves protein docking performance. *Proteins: Structure, Function, and Bioinformatics*, 72(1):270–279, 2008.

[7] Brian G Pierce, Kevin Wiehe, Howook Hwang, Bong-Hyun Kim, Thom Vreven, and Zhiping Weng. ZDOCK server: interactive docking prediction of protein–protein complexes and symmetric multimers. *Bioinformatics*, 30(12):1771–1773, 2014.

[8] Brian Pierce and Zhiping Weng. ZRANK: reranking protein docking predictions with an optimized energy function. *Proteins: Structure, Function, and Bioinformatics*, 67(4):1078–1086, 2007.

[9] Shruthi Viswanath, DVS Ravikant, and Ron Elber. Improving ranking of models for protein complexes with side chain modeling and atomic potentials. *Proteins: Structure, Function, and Bioinformatics*, 81(4):592–606, 2013.

[10] DVS Ravikant and Ron Elber. Energy design for protein-protein interactions. *The Journal of chemical physics*, 135(6), 2011.

[11] DVS Ravikant and Ron Elber. Pie—efficient filters and coarse grained potentials for unbound protein–protein docking. *Proteins: Structure, Function, and Bioinformatics*, 78(2):400–419, 2010.

[12] Carles Pons, David Talavera, Xavier De La Cruz, Modesto Orozco, and Juan Fernandez-Recio. Scoring by intermolecular pairwise propensities of exposed residues (SIPPER): a new efficient potential for protein- protein docking. *Journal of chemical information and modeling*, 51(2):370–377, 2011.

[13] Cyril Dominguez, Rolf Boelens, and Alexandre MJJ Bonvin. HADDOCK: a protein-protein docking approach based on biochemical or biophysical information. *Journal of the American Chemical Society*, 125(7):1731–1737, 2003.
